# β-Catenin and TCFs/LEF signaling discordantly regulate IL-6 expression in astrocytes

**DOI:** 10.1186/s12964-020-00565-2

**Published:** 2020-06-16

**Authors:** KaReisha F. Robinson, Srinivas D. Narasipura, Jennillee Wallace, Ethan M. Ritz, Lena Al-Harthi

**Affiliations:** 1 Rush University Medical Center, Department of Microbial Pathogens and Immunity, Rush University Medical College, 1735 W. Harrison Street, 614 Cohn, Chicago, IL 60612 USA; 2 Rush Biostatistics Core, Rush University Medical College, Chicago, IL USA

**Keywords:** Astrocytes, β-Catenin, TCFs/LEF, IL-6, Neuroinflammation

## Abstract

**Background:**

The Wnt/β-catenin signaling pathway is a prolific regulator of cell-to-cell communication and gene expression. Canonical Wnt/β-catenin signaling involves partnering of β-catenin with members of the TCF/LEF family of transcription factors (TCF1, TCF3, TCF4, LEF1) to regulate gene expression. IL-6 is a key cytokine involved in inflammation and is particularly a hallmark of inflammation in the brain. Astrocytes, specialized glial cells in the brain, secrete IL-6. How astrocytes regulate IL-6 expression is not entirely clear, although in other cells NFκB and C/EBP pathways play a role. We evaluated here the interface between β-catenin, TCFs/LEF and C/EBP and NF-κB in relation to IL-6 gene regulation in astrocytes.

**Methods:**

We performed molecular loss and/or gain of function studies of β-catenin, TCF/LEF, NFκB, and C/EBP to assess IL-6 regulation in human astrocytes. Specifically, siRNA mediated target gene knockdown, cDNA over expression of target gene, and pharmacological agents for regulation of target proteins were used. IL-6 levels was evaluated by real time quantitative PCR and ELISA. We also cloned the IL-6 promoter under a firefly luciferase reporter and used bioinformatics, site directed mutagenesis, and chromatin immunoprecipitation to probe the interaction between β-catenin/TCFs/LEFs and IL-6 promoter activity.

**Results:**

β-catenin binds to TCF/LEF to inhibits IL-6 while TCFs/LEF induce IL-6 transcription through interaction with ATF-2/SMADs. β-catenin independent of TCFs/LEF positively regulates C/EBP and NF-κB, which in turn activate IL-6 expression. The IL-6 promoter has two putative regions for TCFs/LEF binding, a proximal site located at -91 nt and a distal site at -948 nt from the transcription start site, both required for TCF/LEF induction of IL-6 independent of β-catenin.

**Conclusion:**

IL-6 regulation in human astrocytes engages a discordant interaction between β-catenin and TCF/LEF. These findings are intriguing given that no role for β-catenin nor TCFs/LEF to date is associated with IL-6 regulation and suggest that β-catenin expression in astrocytes is a critical regulator of anti-inflammatory responses and its disruption can potentially mediate persistent neuroinflammation.

Video Abstract

**Graphical abstract:**

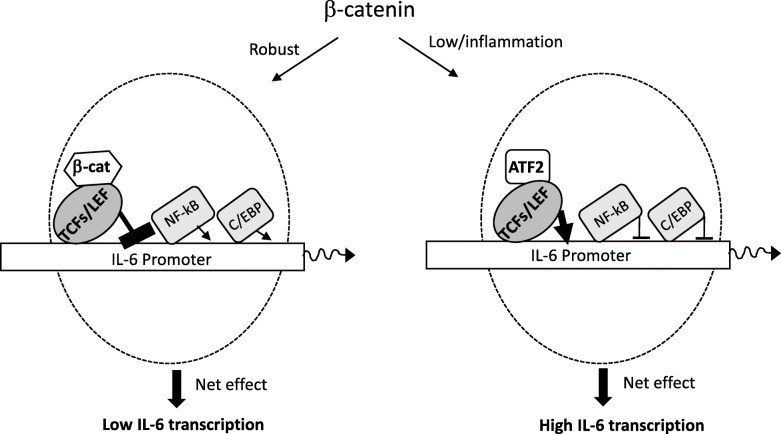

## Background

Inflammation in the Central Nervous System (CNS) is orchestrated by numerous biological factors; among these is the pleiotropic cytokine IL-6. Elevated levels of IL-6 is associated with neuroinflammation/neurodegenerative diseases such as Alzheimer’s (AD) [1–4], Multiple Sclerosis (MS) [5, 6], Parkinson’s Disease (PD) [7, 8], Huntington’s Disease (HD) [9, 10], and HIV-Associated Neurocognitive Disorders (HAND) [11–13] as well as psychiatric disorders such as major depression [14–16] and schizophrenia [17–19]. Most resident CNS cells secrete IL-6, including astrocytes, neurons, microglia, and endothelial cells [20, 21]. Under physiological conditions, low levels of IL-6 are detected in the CNS to support neuronal development, neuronal differentiation, synaptic transmission, and synaptic plasticity [20–22]. In response to injury or infection in the CNS, abundant amounts of IL-6 are produced. Secreted IL-6 causes either inflammation or non-inflammatory effects based on whether IL-6 binds to the membrane bound IL-6 receptor (IL-6Rα) to activate the SHP2-MAPK or JAK-STAT signaling pathways or to the soluble form of the receptor for trans-signaling (enabling cells without IL-6Rα to respond to IL-6) [20, 21, 23]. To add to the complex signaling behavior of IL-6, in MS IL-6 induces proliferation and differentiation of T cells into the CNS by upregulating V-CAM1 [5, 6]. Similarly in HAND higher IL-6 levels are associated with neuronal damage and/or death [11–13]. However in AD, although IL-6 is found to be in areas of beta-amyloid [1–4], it has also been found to have a beneficial role by reducing beta-amyloid deposition and inducing plaque clearance [24]. In HD and PD IL-6 decreases neuronal death by inhibiting Ca^2+^ and ROS excitotoxicity [7–10]. Murine studies have shown that IL-6 causes microgliosis and astrogliosis when overexpressed [25–28] while IL-6 knockout (KO) models have the opposite effect [29–33]. Regulation of IL-6 is likely tightly controlled to prevent hyper-activation within the CNS while balancing the need for neuroimmune communication to elicit an appropriate biological response. Though regulation of IL-6 secretion is unclear, both C/EBP and NF-κB are implicated in the regulation of IL-6 [34–37]. C/EBP is a family of transcription factors with C/EBP-β and C/EBP-δ involved in neuroinflammation [36]. NF-κB is a proinflammatory signaling pathway that drives expression of proinflammatory cytokines such as IL-1 and IL-6 and when elevated, these cytokines are linked to neuropathology. C/EBP and NF-κB family members can heterodimerize and in some cases mediate inflammation [34, 38].

Astrocytes are highly specialized cells in the CNS with well-established immune and non-immune functions including glutamate uptake, maintenance of the blood brain barrier (BBB), and secretion of immune modulators, including IL-6 [20, 39–45]. We previously reported that astrocytes have robust expression of the Wnt/β-catenin signaling pathway and its disrupting, in response to inflammation and or viral infection, leads to dysregulation of key functions of astrocytes including induction of astrocyte senescence [41, 46]. IL-6 is a hallmark cytokine associated with the senescence associated secretory phenotype (SASP), a collection of cytokines and other secretory factors secreted under cellular senescence [47–51]. We therefore assessed the molecular regulation of IL-6 in astrocytes and whether IL-6 is regulated by the Wnt/β-catenin pathway in astrocytes.

β-catenin has two primary functions, a transcriptional co-activator binding to members of the TCFs/LEF family (TCF1, TCF3, TCF4, or LEF1) to regulate gene expression and a component of the adherens junction regulating cell-to-cell communication [44]. TCFs/LEF are differentially expressed in cells and tissues [52–54]. TCF1 (encoded by the TCF7 gene) was the first TCF/LEF family member discovered along with LEF1 (encoded by the LEF1 gene) [53, 55, 56]. TCF1 can act as either a transcriptional activator or repressor, depending on cell and tissue type [57–59]. LEF1 is generally known as an activator of transcription [56, 60]. TCF3 (encoded by the TCF7L1 gene) is the most abundant TCFs/LEF member and is generally known as a repressor for transcription [60–64]. TCF4 (encoded by the TCF7L2 gene) is the most extensively studied member of the TCF/LEF family because it is more ubiquitously expressed in many human adult tissues in comparison to TCF1, TCF3, and LEF1 [65]. Like TCF1, TCF4 can act as either a transcriptional activator or repressor depending on cell and tissue type [58, 59, 66, 67]. Astrocytes are unique in that they express all TCFs/LEF members [44] and robustly express β-catenin [44]. The Wnt/β-catenin signaling pathway is an important neuroprotective pathway and its dysregulation is associated with a number of neurodegenerative diseases, including AD, PD, HAND, and psychiatric disorders such as bipolar disorder and depression [46, 68, 69]. This pathway is vital to various functions in the CNS ranging from memory consolidation in astrocytes, neurogenesis, neurotransmitter release, induction of long-term potentiation and depolarization resulting in increased synaptic strengths [46, 70, 71].

We assessed here the direct impact of β-catenin on IL-6 gene expression in astrocytes. We reveal a complex mechanism where by the effect of β-catenin on IL-6 is discordant with TCFs/LEF. Typically, β-catenin complexes with TCFs/LEF to regulate gene expression. Here, we show that β-catenin inhibits IL-6 promoter activity while also inducing NF-κB and C/EBP, two activators of IL-6. TCFs/LEF on the other hand induce IL-6 gene expression. This complex regulation of IL-6 expression in the CNS underscore its tight regulation as high and/or unregulated IL-6 levels can be neuroinflammatory.

## Materials and methods

### Cell culture and reagents

Normal Human Astrocytes (NHAs) (Lonza, Walkersville, MD) and U138MG astrocytoma cell line (ATCC; Manassas, VA) were maintained and cultured as previously described [72]. Briefly, NHAs were propagated in astrocyte basal media (ABM, Lonza) supplemented with 0.3% heat-inactivated fetal bovine serum (HI-FBS), 30 μL/mL ascorbic acid, 1 μL/mL rhEGF, 1 μL/mL GA-1000 (30 μg/mL gentamicin and 15 μg/mL amphotericin), 2.5 μL/mL insulin, and 10 μL/mL L-glutamine. Passages 1–6 were used in these experiments. U138MG cells were propagated in Dulbecco’s modified eagle’s medium (DMEM; ThermoFisher, Waltham, MA) supplemented with 10% HI-FBS serum (Sigma, St. Louis, MO) and 1% penicillin-streptomycin (ThermoFisher, Waltham, MA). Cells were maintained in a 5% CO_2_ humidified atmosphere at 37 °C. 6-bromoindirubin-3′-oxime (BIO, lot # 025M4611V) and LiCl (lot #: 0001402019) were purchased from Sigma (St. Louis, MO) and re-suspended in appropriate vehicle and stored per manufacturer’s instructions. Human recombinant protein IL-1β (IL038, lot #: 2914570) was purchased from Sigma and re-suspended in appropriate vehicle and stored per manufacturer’s instructions. Small molecule inhibitors of Smads, LDN-193189 (lot #: S261803) and SB525334 (lot #: S147602) were purchased from Selleckchem (Houston, TX) and re-suspend in appropriate vehicle and stored per manufacturer’s instructions. A custom-made ELISA kit (HSTCMAG-28SK, Millipore, Darmstadt, Germany) was designed to measure IL-6 and TNF-α analytes. All solutions, quality controls, and standards were prepared according to the manufacturer’s protocol and the data was acquired and analyzed on the FLEXMAP 3D machine (Luminex Corp, Northbrook, IL). Firefly luciferase assay system was purchased from Promega (E1500, Madison, WI) and analyzed using the Sirius Single tube luminometer (Berthold Detection Systems, Pforzheim, Germany).

### Bioinformatic search for TCFs/LEF DNA binding sequences in IL-6 promoter

The locus of IL-6 gene on human genome was identified by searching on PubMed. Approximately 1.4 kb region upstream of transcription start site was identified as putative promoter region. The promoter elements such as CRE, C/EBP, NF-κB, and AP-1 previously characterized for IL-6 [73, 74] were all found to be present in the putative promoter sequence. The Sequencher program, software version 5.4.6 (Genes Code incorporation, Ann Arbor, MI) was used to locate TCFs/LEF binding sites on the IL-6 promoter using the TCFs/LEF consensus sequence 5′-CAAAGA-3′.

### Plasmid construction and site-directed mutagenesis

Genomic DNA was obtained from cultured astrocytes using a QIAampDNA Mini and Blood Mini kit (Qiagen, Hilden, Germany). The promoter region of IL-6 was amplified using the primers SN282 (F-5′-GAGAGGTACCTGTGCAAGGGTCTGGTTTC-3′) and SN283 (R-5′-GAGACTCGAGGATAGAGCTTCTCTTTCGTTCCC-3′) cut with KpnI and XhoI (respective underlined sequences) (New England Biolabs, Ipswich, MA) and purified with PCR column purification (Qiagen). The following PCR conditions were used: initial denaturation at 95 °C for 10 min.; another denaturation at 95 °C for 30 s, annealing at 60 °C for 30 s, and extension at 72 °C for 1.5 min for 35 cycles; and another extension at 72 °C for 10 min using the AmpliGold Taq polymerase. The IL-6 insert was cloned into XhoI and KpnI predigested, PCR column purified pGL4.12 vector (p501) (Promega, Madison, WI) by ligation followed by bacterial transformation. Recombinant plasmid was subjected to restriction digestion with KpnI and XhoI and sequencing to confirm the presence of the insert. WT-IL-6 promoter reporter plasmid (p563B) was subjected to site-directed mutagenesis using the Q5 Site-Directed Mutagenesis Kit (New England Biolabs) to obtain the substitution at the distal TCFs/LEF binding site, p573 (primers KR1, F-5’ACCCTCCAACctgGATTTATCAAATGTGGGATTTTCCCATGAGTCTC-3′ and KR2, R-5′- GAGGGTGGGGCCAGAGCG-3′) and the substitution at the proximal TCFs/LEF binding site, p575 (primers KR5, F-5′-CATCCCCAACctgGAGGTGAGTAG-3′ and KR6, F-5′-TCAAAGGAGGACCTTGTG-3′). Both primer sets were used to make the double mutant substitution at both TCFs/LEF binding sites, p578.

### Plasmid and siRNA transfections

siRNAs at 100 nm concentration were transfected into NHAs using Lipofectamine RNAimax as per manufacturer’s instructions (Invitrogen, Carlsbad, CA). ON-TARGET plus SMARTpool siRNAs specific for TCF1 (L-019735-00), TCF3 (L-014703-00), TCF4 (L-003816-00), LEF1 (L-015396-00), β-catenin (L-003482-00), C/EBP-β (L-006423-00), C/EBP-δ (L-010453-00), RelA (L-003533-00), ATF2 (L-009871-00), Zeb1 (L-006564-01), and scrambled (D-001810-10) were procured from Dharmacon (Lafayette, CO, USA). Cells were approximately 90% confluent at the time of transfection and processed 48-72 h post transfection for further downstream applications. Transfection of plasmids at 0.25 μg/24 well format with or without the indicated siRNAs in NHAs were performed using Lipofectamine 3000 as per manufacturer’s instructions. Transfections of plasmids at 0.25 μg/24 well format in U138s were done using polyethylenimine (PEI, linear) as per manufacturer’s instructions (Sigma, St. Louis, MO). Control pcDNA plasmid (#10792) was obtained from Addgene (Watertown, MA). Cis-acting luciferase reporter plasmids specific for C/EBP (#240112–51) and NF-κB activities (#219078–51) were obtained from Stratagene (San Diego, CA). Expression vector harboring cDNAs of each TCFs/LEF family members were obtained from ABMgood (Richmond, BC, Canada).

### Quantitative real-time PCR

RNA was isolated using the RNeasy Mini Kit (Qiagen, Hilden, Germany), according to the protocol. RNA was digested with DNase I (Sigma, St. Louis, MO) for 15 min at RT to remove DNA. Subsequently, DNase I was inactivated by heating at 70 °C for 15 min. cDNA was synthesized using Qscript supermix (Quanta Biosciences, Beverly, MA). Real-time PCR was performed using SSO fast SYBR green supermix (Biorad, Hercules, CA) in a 7500 real-time PCR system (Applied Biosystems, Waltham, MA) using 7500 software v2.0.1. Melting curve analysis was performed to ensure the amplification of a single product. Primers used are: β-catenin-F-5′-TCTTGCCCTTTGTCCCGCAAATCA-3′ and β-catenin-R-5′-TCCACAAATTGCTGTGTCCCA-3′; IL-6-F-5′-GGAGACTTGCCTGGTGAAA-3′ and IL-6-R-5′-CTGGCTTGTTCCTCACTACTC-3′; TCF1-F-5′-AGGCCAAGAAGCCAACCATCAAGA-3′ and TCF1-R-5′-ACTCTGCAATGACCTTGGCTCTCA-3′; TCF3-F-5′-TGCAGTGAGCGTGAAATCACCAGT-3′ and TCF3-R-5′-AATGGCTGCACTTTCCTTCAGGGT-3′; TCF4-F-5′-TCGGCAGAGAGGGATTTAGCTGATGT-3′ and TCF4-R-5′-CTTTCCCGGGATTTGTCTCGGAAACT-3′; LEF1-F-5′-AAGCATCCAGATGGAGGCCTCTACAA-3′ and LEF1-R-5′-TGATGTTCTCGGGATGGGTGGAGAAA-3′; C/EBP-α-F-5′-GAAGTCGGTGGACAAGAACA-3′ and C/EBP-α-R-5′-TCATTGTCACTGGTCAGCTC-3′; C/EBP-β-F-5′-CGCGACAGGGCCAAGAT-3’and C/EBP-β-R-5′-GCTGCTCCACCTTCTTCTG-3′; C/EBP-δ-F-5′–CATCGACTTCAGCGCCTAC-3′ and C/EBP-δ-R-5′-GCCTTGTGATTGCTGTTGAAG-3′; C/EBP-γ-F-5′-GGATCGAAACAGTGACGAGTAT-3′ and C/EBP-γ-R-5′-GCAGTGTGTCTTGTGCTTTC-3′; C/EBP-ε-F-5′-CAGCTTCTCTCCGATCTCTTTG-3′ and C/EBP-ε-R-5′-GTCAGGCGGCAAGTAGTG-3′; C/EBP-ζ-F-5′-GGAGTTGTGTCTGGTGAAGTAG—3′ and C/EBP-R-5′-TGGGACAGAGCCATTTGATTTA-3′; NF-kB-F-5′-AGGATGAAGGAGTTGTGCCTGGAA-3′ and NF-kB-R-5′-TCAGCCAGCTGTTTCATGTCTCCT-3′; NF-κB1-F-5′-GAGACATCCTTCCGCAAACT-3′ and NF-κB1-R-5′-GGTCCTTCCTGCCCATAATC-3′; NF-κB2-F-5′-GGACTGTCACTTGGTGATACAG-3′ and NF-κB2-R-5′-TGTCTGTCGGTACGTGTCTA-3′; RelA-F-5′-TGGGAATCCAGTGTGTGAAG-3′ and RelA-R-5′-CACAGCATTCAGGTCGTAGT-3′; RelB-F-5′-GAGCCCGTCTATGACAAGAAA-3′ and RelB-R-5′-TTGTCGCAGAGCAAGTAGAG-3′; c-Rel-F-5′-CCCACCATTCCTGAGAATACC-3′ and c-Rel-R-5′- ACGCTTCCATTCCGACTATG-3′; Smad1-F-5′-CAGAAGGAGGTCTGCATCAA-3′ and Smad1-R-5′- GAGGCTGTGCTGAGGATTAT-3′; Smad2-F-5′-GGGACTGAGTACACCAAATACG-3′ and Smad2-R-5′-TACCTGGAGACGACCATCAA-3′; Smad3-F-5′-CCTGAGTGAAGATGGAGAAACC-3′ and Smad3-R-5′-GGCTGCAGGTCCAAGTTATTA-3′; Smad4-F-5′-TCCAGCATCCACCAAGTAATC-3′ and Smad4-R-5′-GCAGTGCTGGTAGCATTAGA-3′; Smad5-F-5′-CTATGTTGGTGGAGAGGTGTATG-3′ and Smad5-R-5′-CAGACAGTGGTGGGATGAAA-3′; Smad6-F-5′-GAATTCTCAGACGCCAGCAT-3′ and Smad6-R-5′- TGGTCGTACACCGCATAGA-3′; Smad7-F-5′-CTCCATCAAGGCTTTCGACTAC-3′ and Smad7-R-5′-AGCTGATCTGCACGGTAAAG-3′; Smad8/9A-F-5′-AGCCAGAGAGTCCCTATCAA-3′ and Smad8/9A-R-5′-GTCTATCAGCTGTGGCATCTAC-3′; Smad8/9B-F-5′-TAGGAAAGGGTGTGCACTTG-3′ and Smad8/9B-R-5′-GTGGAAGCCGTGTTGATAGT-3′; ATF2-F-5′- GTCATGGTAGCGGATTGGTTAG-3′ and ATF2-R-5′-CGGAGTTTCTGTAGTGGATGTG-3′; Zeb1-F-5′-CCCAGGACAGCACAGTAAAT-3′ and Zeb1-R-5′-GATGGTGTACTACTTCTGGAACC-3′; ID1-F-5′-CGACATGAACGGCTGTTACTC-3′ and ID1-R-5′-GGTCCCTGATGTAGTCGATGA-3′; PAI-F-5′-CTGGTGAATGCCCTCTACTTC-3′ and PAI-R-5′-TGCTGCCGTCTGATTTGT-3′; and GAPDH-F-5′-TGACTTCAACAGCGACACCCACT-3′ and GAPDH-R-5′-ACCACCCTGTTGCTGTAGCCAAAT-3′. Fold change in mRNA expression was calculated by relative quantification using the comparative C_T_ method with GAPDH as the endogenous control.

### Chromatin Immunoprecipitation (ChIP)

ChIP was performed using the Imprint Chromatin Immunoprecipitation kit (CHP1) protocol (Sigma) with antibodies for β-catenin (anti-Rabbit, Sigma-Aldrich; #C2206), TCFs/LEF Family Antibody Sampler Kit (Cell Signaling, #9383 T), and Rabbit IgG control (Cell Signaling, #3900). Per immunoprecipitation, ~ 1–2 × 10^6^ cells and 4 μg of antibody were used. ChIP DNA was washed and purified as per the manufacturer’s protocol. Samples were analyzed by quantitative real-time PCR as indicated above. Primers used to amplify IL-6 promoter regions were divided into two regions: proximal (F-5′-CCTCACCCTCCAACAAAGATT-3′ and R-5′-CCTCAGACATCTCCAGTCCTAT-3′) and distal (F-5′-AGGGAGAGGGAGCGATAAA and R-5′-ACTTGGTTCAGGGCAGAAAG-3′). Data was normalized to IgG and represented as fold change with respect to IgG.

### Western blot

For western blotting, cells were lysed with radioimmunoprecipitation assay (RIPA) buffer and total protein content was estimated by bicinchoninic acid assay (BCA) (Bio-Rad, Des Plaines, IL). Ten to twenty micrograms of total cell lysate were separated by 10% SDS-PAGE, transferred onto a nitrocellulose membrane, blocked with superblock (ThermoFisher) containing 0.1% Tween 20 (T20) for 1 h, incubated with primary antibody for 1 h at RT for β-catenin (1:10,000; Rabbit; Sigma, #C2206) or Glyceraldehyde 3-phosphate dehydrogenase (GAPDH, Rabbit, Sigma, #G9545) overnight at 4 °C in superblock-0.1% T20. Membranes were washed extensively in Tris-buffered saline-Tween 20 (TBS-T) and incubated with secondary antibody conjugated to horseradish peroxidase (HRP) (1:50,000; Cell Signaling, #7074) in Superblock-0.1% T20 for 45 min. at RT. Membranes were again washed extensively in TBST and developed with SuperSignal West Femto Maximum Sensitivity Substrate (ThermoFisher) according to the instructions. Analyses of band densitometry was quantified using ImageJ software version 1.42q (National Institutes of Health, Bethesda, MD).

### Statistical analysis

Statistical analyses were performed with consultation of Rush statistical core using Prism software (GraphPad Prism, San Diego, CA). The variables were compared using either the two-tailed one sample T-test or the one-way ANOVA with the post hoc Dunnett’s on the data. *p*-value of ≤0.05 was considered significant and all experiments were performed independently at least three times.

## Results

### β-Catenin negatively regulates IL-6 transcription in human astrocytes

**Fig. 1:**
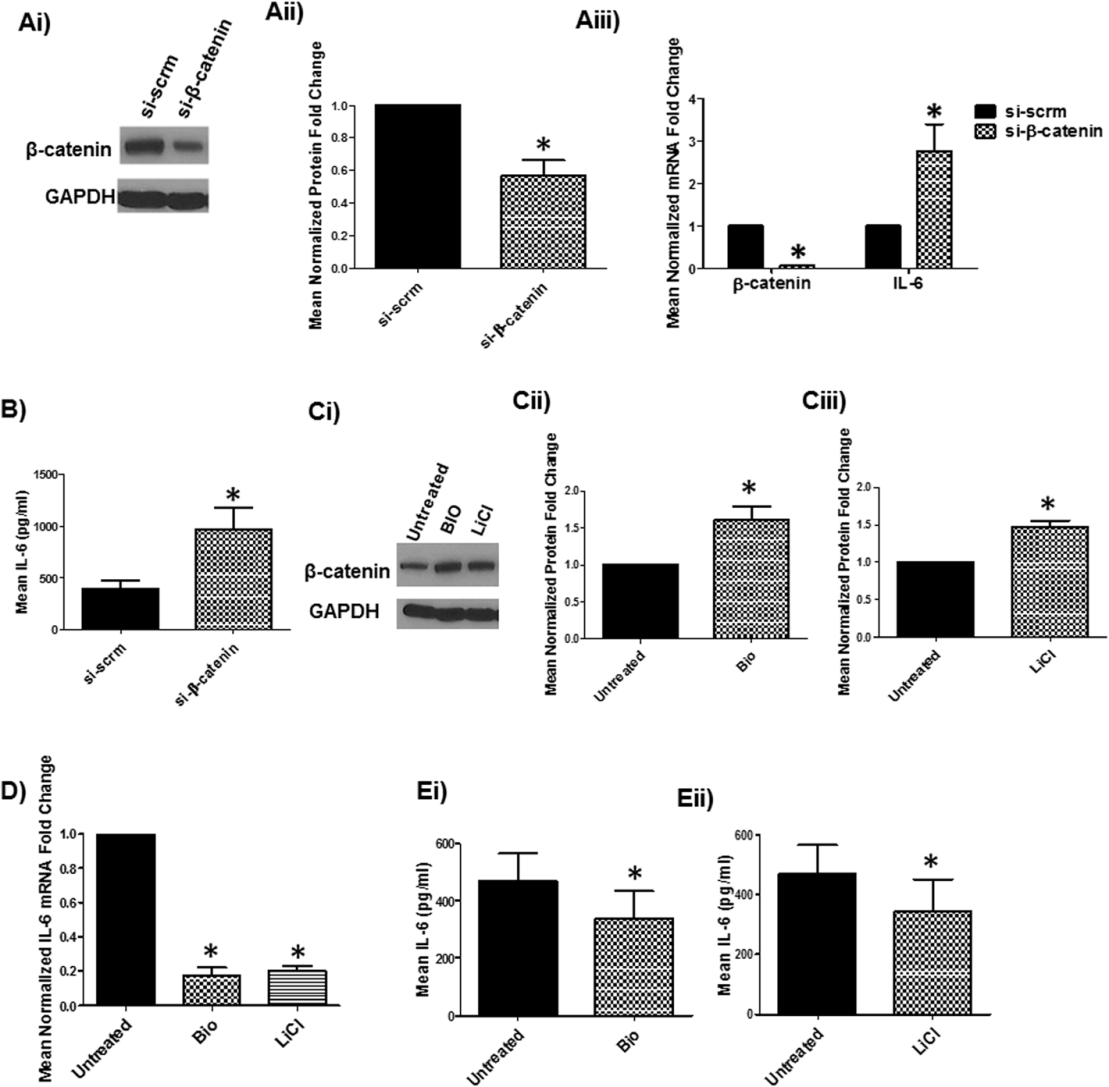
β-catenin negatively regulates IL-6 transcription in normal human astrocytes (NHAs). **a** NHAs were transfected with β-catenin or scrambled (scrm) siRNA and at 48 h, β-catenin and IL-6 mRNA levels were measured by real-time PCR. Efficacy of KD of β-catenin protein is shown by western blot (Ai) and quantified with densitometer (Aii). **b** NHAs were transfected with β-catenin or scrm siRNA and at 48 h IL-6 protein was measured by ELISA. **c** Astrocytes were either treated with activators of β-catenin (BIO at 0.5 μM or LiCl at 5 mM) or left untreated and at 24 h, β-catenin protein was measured by Western Blot (Ci) and quantified by densitometry (Cii-Ciii). Effect of β-catenin activators on IL-6 mRNA (**d**) and IL-6 protein (**e**) were measured by real-time PCR and ELISA, respectively. *indicates *p* ≤ 0.05 in comparison to respective control. The data is presented as mean ± SEM (*n* = ≥3; one sample T-Test)

A previous study from our lab demonstrated that inhibition of β-catenin in human astrocytes leads to astrocyte senescence [41]. Given that IL-6 expression constitutes a Senescence Associated Secretory Phenotype (SASP) [75, 76], we assessed whether β-catenin regulates IL-6 gene expression. Astrocytes express IL-6 mRNA as determined by qPCR (Supl. Figure 1). siRNA knockdown (KD) of β-catenin induced IL-6 mRNA and protein expression by ~ 3–2–fold, respectively (Fig. 1ai and b). Efficiency of β-catenin mRNA KD was consistently greater than 90% (Fig. 1aii). Conversely, induction of β-catenin using two pharmacologic agents (BIO and LiCl) [77, 78] (Fig. 1c) reduced IL-6 mRNA and protein by ~ 75 and 40%, respectively (Fig. 1d and e). Together, these loss- and gain-of-function studies demonstrate that β-catenin negatively regulates IL-6 transcription in astrocytes.

### TCFs/LEF positively regulates IL-6 mRNA expression in human astrocytes

**Fig. 2:**
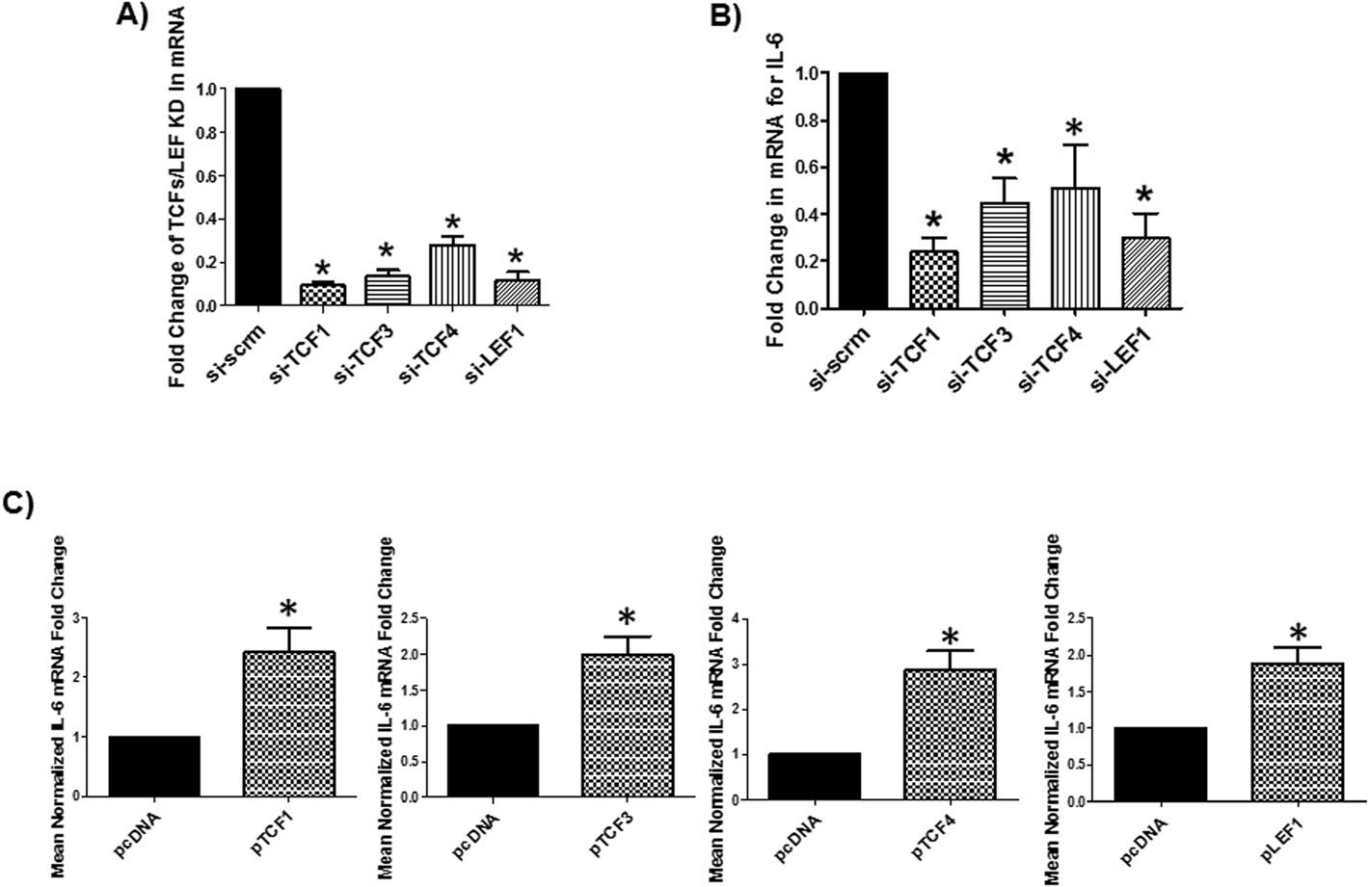
TCFs/LEF positively regulate IL-6 expression in normal human astrocytes. **a** NHAs were transfected with TCF1, TCF3, TCF4, LEF1, or scrambled (scrm) siRNA and at 48 h, TCF1, TCF3, TCF4, and LEF1 mRNA level measured by real-time PCR and data normalized to GAPDH. **b** NHAs were KD for TCF/LEF transcription factors (TFs) and IL-6 mRNA measured at 48 h. by real-time PCR and normalized to GAPDH. **c** NHAs were transfected with TCF1, TCF3, TCF4, LEF1, or pcDNA (control) expression plasmids and at 24 h IL-6 mRNA expression was measured by real-time PCR. * indicates p ≤ 0.05 in comparison to control. The data is presented as mean ± SEM (n = ≥ 3; in (**a**) and (**b**), one-way ANOVA with post hoc Dunnett’s test was performed and in **c** one sample T-Test was performed)

Typically, β-catenin forms a complex with members of the TCFs/LEF family (TCF1, TCF3, TCF4, or LEF1) to regulate gene expression. We evaluated whether TCFs/LEF transcription factors (TFs) also inhibit IL-6 mRNA expression. Human astrocytes express all TCFs/LEF mRNAs to similar levels as the housekeeping gene GAPDH (Suppl. Fig. 2a). To determine which of these TFs is regulating IL-6, we knocked down each TCFs/LEF using siRNAs. The efficacy of KD for TCF1, TCF3, TCF4 and LEF1 was ≥78% as measured by qRT-PCR (Fig. 2a). Importantly, KD of individual TCFs/LEF family member did not affect the expression of other TCF/LEF TFs, indicating that the targeted siRNAs were specific to the TCFs/LEF family member (Suppl. Fig. 2b). KD of TCF1, TCF3, TCF4, or LEF1 all significantly reduced IL-6 mRNA by > 50% (Fig. 2b). Conversely, transfecting the cells with cDNA plasmids for TCF1, TCF3, TCF4, LEF1 or pcDNA (control vector) induced IL-6 mRNA by ~ 3-fold (*p* ≤ 0.05, Fig. 2c). These data demonstrate that TCFs/LEF TFs are positive regulators of IL-6 gene expression while β-catenin is a negative regulator, highlighting a discordance between β-catenin and TCFs/LEF effect on IL-6 gene regulation.

### Identification of functional TCFs/LEF binding sites on the IL-6 promoter

**Fig. 3:**
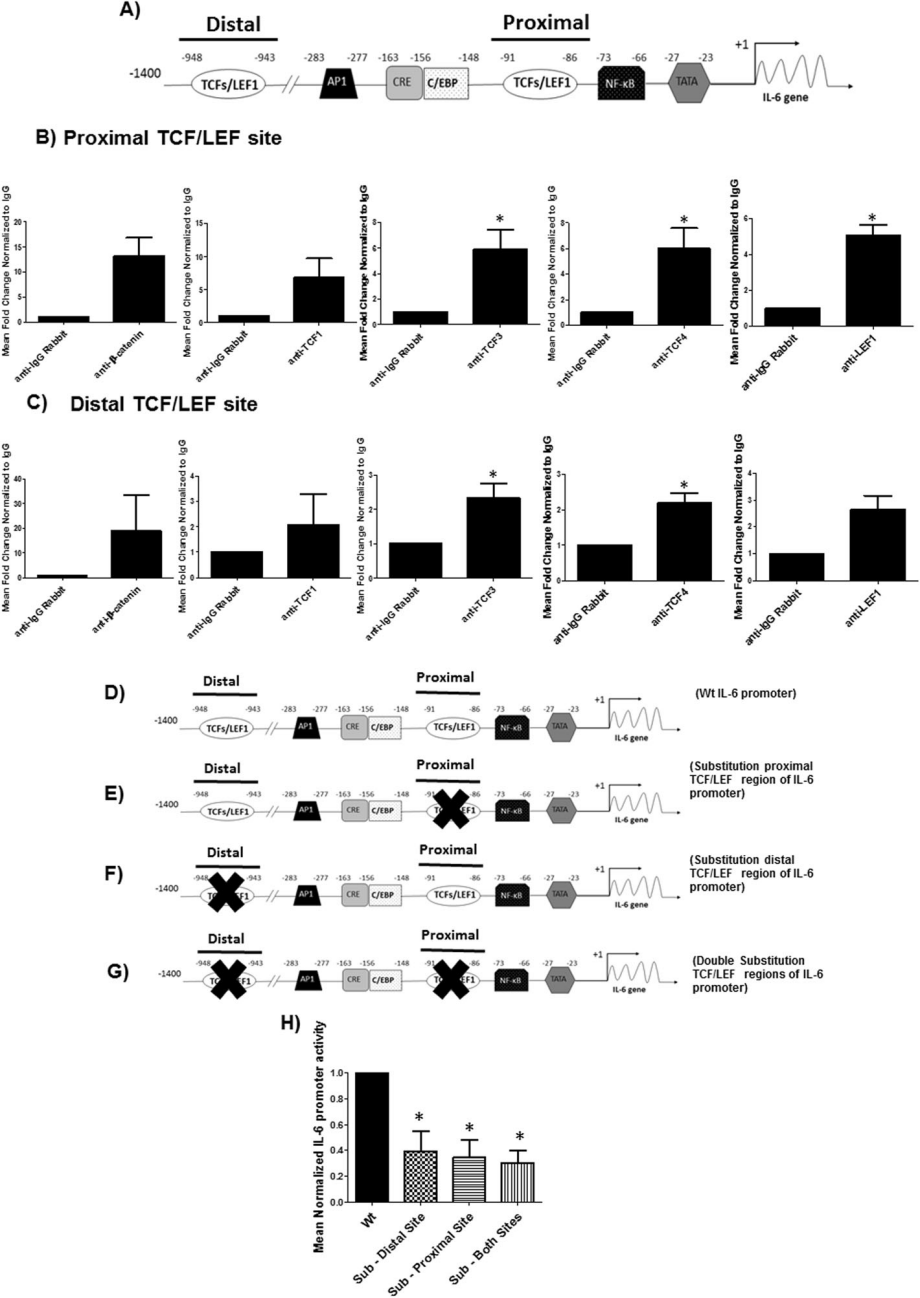
Identification of functional binding sites of TCFs/LEF and preferential tethering of TCF/LEF family members on IL-6 promoter. **a** Identification of two putative TCF/LEF sites located between − 91 and − 86 (1st site; proximal) and − 948 and − 943 (2nd site; distal) on the IL-6 promoter by bioinformatic analysis. **b** and **c** ChIP was performed using 4 μg of β-catenin, TCF1, TCF3, TCF4, LEF1, or isotype IgG control antibodies and DNA amplified spanning the proximal (**b**) or distal (**c**) sites on the IL-6 promoter. *indicates *p* ≤ 0.05 in comparison to isotype IgG control (*n* = 5 for all and *n* = 2 for β-catenin ChIP , one sample T-Test). **d** Wild-type **(**wt) IL-6 reporter plasmid was mutated by substitution on the proximal (**e**), distal (**f**), or both (**g**) TCF/LEF putative binding sites. **h** NHAs were transfected with either the wt IL-6 promoter plasmid or the mutated plasmids shown in (**d**), (**e**), and (**f**). At 48 h post transfection, IL-6 promoter activity was measured through luciferase reporter assay. * indicates p ≤ 0.05 in comparison to IL-6 wt. The data is presented as mean ± SEM (*n* = 5, one-way ANOVA, post hoc Dunnett’s)

Given the discordance between β-catenin and TCFs/LEF effects on IL-6 gene transcription, we probed the molecular mechanism by which TCFs/LEF positively regulates IL-6 mRNA. Using bioinformatics analyses, we identified two putative regions for TCFs/LEF binding (CAAAGA) in the human IL-6 promoter, a proximal site located at -91 nt and a distal site at -948 nt from the transcription start site (Fig. 3a). We then amplified and cloned this entire 1.4 kb putative IL-6 promoter region into the pGL4.12 luciferase reporter vector. To determine whether the insert is a promoter element for luciferase gene and whether this activity was specific to IL-6 gene expression, we transfected human astrocytes with pGL4.12 (p501, vector control) and the IL-6 promoter reporter plasmid (p563B). Twenty-four hour post-transfection, the cells were either stimulated or unstimulated with an inducing agent specific for IL-6 expression (125 ng/mL IL-1β and luciferase activity was measured 24 h post treatment. The IL-6 insert functioned as a promoter for the pGL4.12 vector under basal conditions (Suppl. Fig. 3a) and stimulation with IL-1β significantly induced IL-6 promoter activity (Suppl. Fig. 3b). These studies demonstrate that putative DNA binding sites for TCFs/LEF within IL-6 promoter is functional to drive promoter expression and is inducible (Suppl. Fig. 3a and b). Knockdown of β-catenin in cultures transfected with the wild type IL-6 reporter plasmid containing sites for TCFs/LEF DNA binding induced IL-6 reporter activity by 1.5-fold (Suppl. Fig. 3c). This data further confirms that β-catenin inhibits IL-6 at the transcriptional level.

### Preferential tethering of TCF/LEF family members on IL-6 promoter

We performed chromatin immunoprecipitation (ChIP) assays to assess whether β-catenin and/or TCFs/LEF are tethered on the distal and/or proximal TCFs/LEF sites within the IL-6 promoter. Using antibodies for β-catenin, TCF1, TCF3, TCF4, LEF1 or isotype IgG control, followed by qPCR for endogenous IL-6 promoter region encompassing both proximal and distal TCFs/LEF binding sites (Fig. 3a), we show that TCF3 and TCF4 are bound at both the proximal and distal TCF/LEF sites while LEF1 was bound only at the proximal site. β-catenin exhibited strong binding affinity on both sites (10–30 fold, respectively) (Fig. 3b and c). TCF1 was not bound at either site at a statistically significant level in comparison to IgG.

### TCFs/LEF sites at the proximal and distal sites are important for IL-6 promoter activity

To assess whether the proximal and/or distal TCFs/LEF sites (Fig. 3d) on the IL-6 promoter regulates basal and inducible IL-6 activity, we performed site-directed mutagenesis. We generated three constructs with substitution (CAAAGA → CCTGGA) at the proximal site (Fig. 3e), distal site (Fig. 3f), or at both the proximal and distal binding sites (Fig. 3g). Endogenous IL-6 reporter activity was significantly reduced by mutating the distal site, the proximal site, and mutations at both site by ~ 70% (Fig. 3h). These data demonstrate that both proximal and distal TCFs/LEF sites are essential for IL-6 promoter activity.

### TCFs/LEF induction of IL-6 is independent of β-catenin and depends on other transcriptional co-regulators (ATF2 and SMADS)

**Fig. 4:**
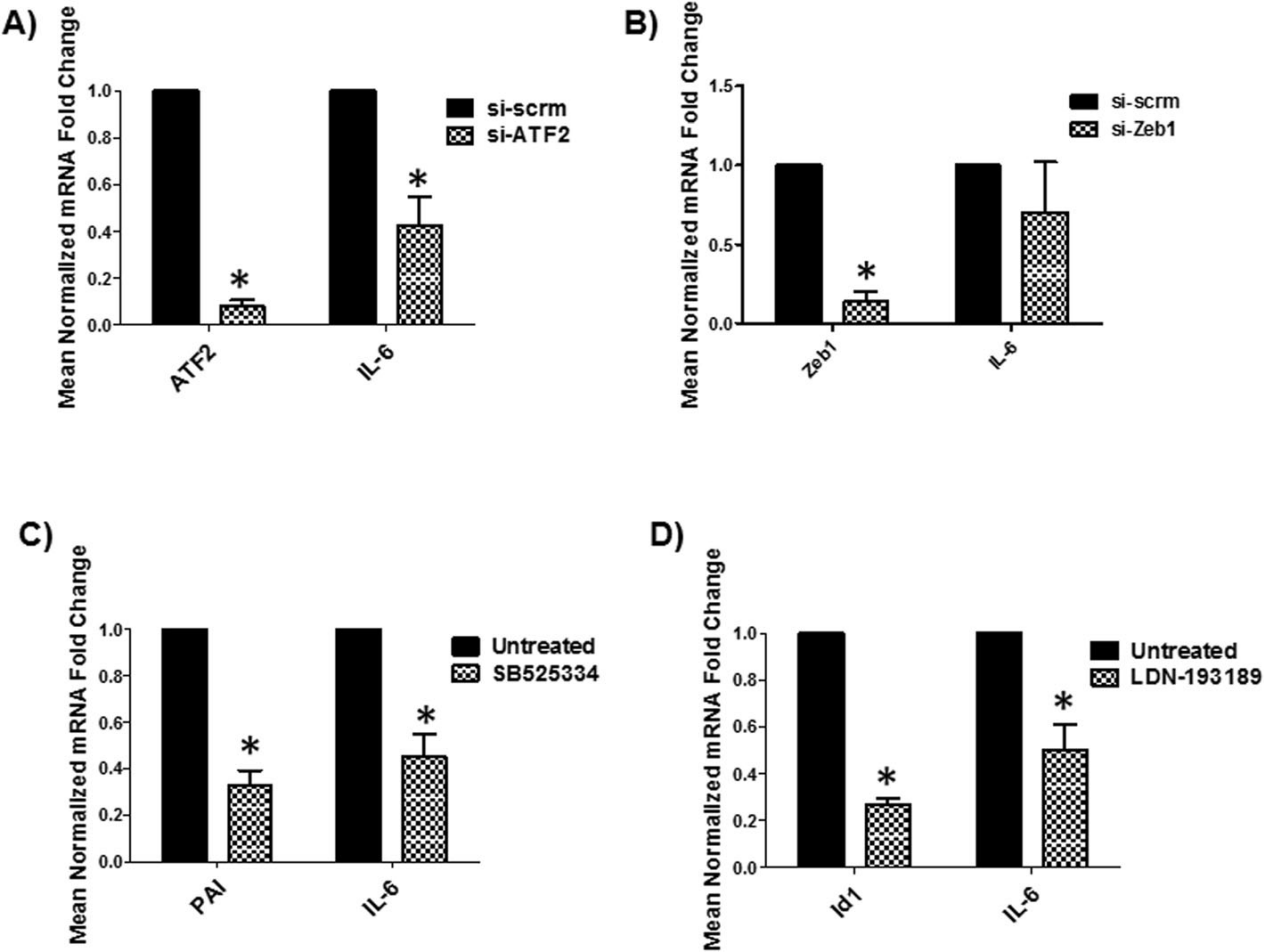
Role of TCF/LEF alternative transcriptional partners (ATF2 and Smads) in IL-6 gene regulation. **a** NHAs were transfected with siRNA for ATF2, Zeb1, or scrambled (scrm) siRNA (**a**, **b**, respectively) or treated with Smads 2 and 3 inhibitor (SB525334) or Smads 1, 5, and 8/9 inhibitor (**c** and **d** respectively). Target gene as indicated was measured at 48 h by real-time PCR in the siRNA KD experiments (**a**, **b**) and at 24 h post small molecule inhibitor experiments (**c**, **d**). * indicates *p* ≤ 0.05 in comparison to control. The data is presented as mean ± SEM (*n* = 3, one-way ANOVA, post hoc Dunnett’s performed in (**a**), (**b**) and one sample T-test performed in (**c**) and (**d**))

Canonical β-catenin signaling involves the binding of β-catenin to TCFs/LEF family members to regulate gene expression. Our data indicate that β-catenin and TCFs/LEF have a discordant impact on IL-6 gene regulation. Specially, β-catenin inhibits while TCFs/LEF induces IL-6 transcription. To determine which transcriptional co-regulators may be involved in TCFs/LEF induction of IL-6, we assessed the role of Activating Transcription Factor 2 (ATF2), *Caenorhabditis elegans* SMA (“small” worm phenotype) and Drosophila MAD (“Mothers Against Decapentaplegic”) family of genes (SMADs), and Zinc finger E-box binding homeobox 1 (ZEB1) in induction of IL-6. All three can partner with TCFs/LEF to regulate gene expression independent of β-catenin [79–85]. We show that human astrocytes express ATF2, SMADs, and ZEB1 to levels relative to GAPDH (Suppl. Fig. 4). To determine which of these co-factors may also be regulating IL-6, we either knocked them down individually using siRNAs or treated the cells with small molecule inhibitors. SB525334 is a small molecule inhibitor for Smads 2 and 3 and LDN-193189 is a small molecule inhibitor for Smads 1, 5, and 8/9. Efficacy of KD for ATF2 and ZEB1 was measured by qPCR and was consistently ≥85% (Figs. 4a and b). Efficacy of small molecule inhibitor was assessed by measuring their effect on their respective target genes. A downstream target of Smads 2 and 3 is Plasminogen activation inhibitor (PAI) while inhibitor of DNA binding protein (Id1) is a downstream target of Smads 1, 5, and 8/9 (Figs. 4c and d). Knockdown of ATF2 reduced IL-6 mRNA by > 50% (Fig. 4a). Knockdown of ZEB1 had no effect on IL-6 mRNA (Fig. 4b). Both SB525334 and LDN-193189 inhibited IL-6 transcription by ~ 65 and ~ 50%, respectively (Fig. 4c and d). These results show that ATF2 and Smads 1, 2, 3, 5, 8/9A and 8/9B all positively regulate IL-6 expression in astrocytes and likely do so by partnering with TCF/LEF family members.

### β-Catenin independent of TCFs/LEF positively regulates C/EBP and **NF-κB**

**Fig. 5:**
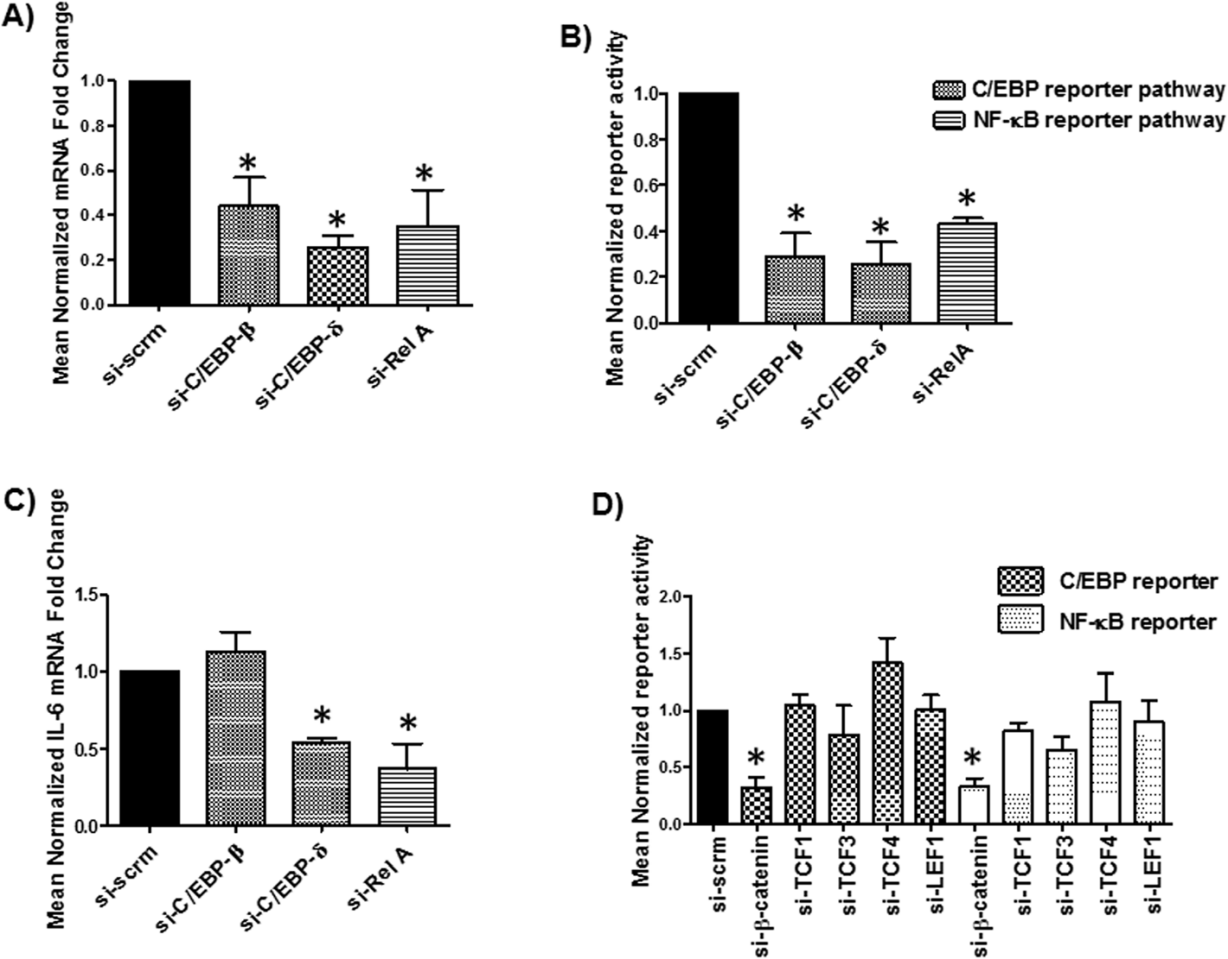
Inhibition of β-catenin downregulates C/EBP and NF-κB activity in NHAs. **a** NHAs were transfected with C/EBP-β, C/EBP-δ, RelA, or scrm siRNA and at 48 h, their respective mRNA expression was measured by real-time PCR. **b** NHAs were transfected with C/EBP-β, C/EBP-δ, Rel A, or scrm siRNA and at 24 h co-transfected with C/EBP or NF-κB reporter plasmids and 24 h later luciferase reporter activity was measured. **c** NHAs were transfected with C/EBP-β, C/EBP-δ, RelA, or scrm siRNA and at 48 h IL-6 mRNA expression was measured by real-time PCR. **d** NHAs were transfected with β-catenin, TCF1, TCF3, TCF4, LEF1, and scrm siRNA for 24 h then transfected with C/EBP or NF-κB reporter plasmid for another 24 h followed by luciferase reporter activity measurement at 48 h post transfection. * indicates *p* ≤ 0.05 in comparison to respective control. The data is presented as mean ± SEM (*n* = ≥3, one-way ANOVA, post hoc Dunnett’s)

C/EBP and NF-κB pathways are well documented to positively regulate IL-6 expression [34, 36, 86–88]. Given that β-catenin inhibition of IL-6 gene is not likely to be driven by its association with IL-6 promoter (e.g. β-catenin is not tethered on the IL-6 promoter, Figs. 3b and c), we assessed the interface between β-catenin, TCFs/LEF and C/EBP and NF-κB in relation to IL-6 gene regulation. Human astrocytes express all NF-κB family members (RelA, RelB, NF-κB1, NF-κB2, c-Rel) and all C/EBP family members (−β, −δ, −γ, −ζ) except α and ε (Suppl. Fig. 5). C/EBP-β, C/EBP-δ, or RelA siRNAs were effective in inhibiting both their respective target gene and respective C/EBP and NF-κB reporter plasmids activity (Figs. 5a and b). KD of C/EBP-δ and Rel A reduced IL-6 mRNA by 50 and 60%, respectively (Fig. 5c), whereas KD of C/EBP-β had no effect on IL-6 expression. Further, β-catenin KD reduced C/EBP and NF-κB reporter activities by ~ 70% while knockdown of TCFs/LEF had no effect on C/EBP or NF-κB activities (Fig. 5d). These findings demonstrate that β-catenin independent of TCFs/LEF positively regulates C/EBP and NF-κB, which in turn are known to activate IL-6 expression.

## Discussion

Astrocytes secrete a number of cytokines and their regulation is likely to be tightly coordinated and regulated as their over-expression may lead to inflammation in the CNS, which is highly neurotoxic. We assessed here the molecular mechanism by which astrocytes regulate IL-6 gene expression. We show that IL-6 regulation at the transcriptional level is indeed complex. β-catenin partners with TCF/LEF at two newly identified sites on the IL-6 promoter to inhibit IL-6 expression, yet it also induces NF-κB and C/EBP, two factors well established to induce IL-6 promoter activity [35, 89, 90]. Likewise, TCFs/LEF family members, which typically partner with β-catenin, function independently of β-catenin to induce IL-6 transcription through partnering with ATF2 and SMAD family members. Taken together, these data demonstrate that atypical activity of β-catenin and TCFs/LEF regulate IL-6 gene expression in astrocytes.

Astrocytes express robust level of canonical Wnt/β-catenin signaling and express all of the TCFs/LEF family members, making astrocytes an ideal cell type to study canonical Wnt/β-catenin signaling [43, 44, 91]. Our working model informed by published [92–94] and data presented here indicate that under homeostatic conditions, where there is robust β-catenin expression, β-catenin induces NF-κB and C/EBP to induce IL-6 promoter activity. This induction can be viewed as a “normal” level of IL-6 that is present in the CNS under normal conditions to promote myelination of oligodendrocyte, synaptic transmission, synaptic plasticity, and overall cell survival. However, under non-homeostatic conditions (e.g. inflammation), β-catenin level is reduced [41–43, 91, 95], as such TCFs/LEF can now partner with factors other than β-catenin. Here, it is ATF2 and members of the SMAD family, to induce IL-6 expression. The level may potentially be higher than what is typically found under physiologic conditions and as such, the increased IL-6 expression can lead to heightened inflammation. High IL-6 level is a hallmark feature of a number of neurodegenerative diseases including MS, AD, PD and brain tumors [20].

Our studies further demonstrate that TCFs/LEF can function independently of β-catenin to regulate gene expression. β-catenin typically partners with one member of TCFs/LEF to regulate gene expression. For example, we previously demonstrated that β-catenin partners with TCF4 to inhibit HIV replication at the transcriptional level in astrocytes [43], it partners with TCF1 to induce Excitatory Amino Acid Transporter 2 (EAAT2) gene expression, and it partners with TCF3 to induce Glutamine Synthetase (GS) gene expression [72]. Here, we show discordant β-catenin and TCFs/LEF effects on IL-6 promoter activity. Interestingly, any of the TCFs/LEF family members can induce IL-6 expression and they may do so as a complex as knocking one member down did not interfere with gene expression of the other members. While all TCF3, TCF4, and LEF1 were found to be tethered on IL-6 promoter either at both proximal and distal site or only at distal site, TCF1 tethering on IL-6 promoter did not reach statistically significant levels beyond IgG isotype control. This could be that its binding is low to be significant, although in some experiments higher binding was trending. Limited studies demonstrate the ability of TCFs/LEF to function as a complex to regulate gene expression. In one study, TCF4 and LEF1 were reported to form a complex to regulate gene expression [96]. More often than not, TCFs/LEF family members exert complementary roles. For example, a TCF1/LEF1 double knockout murine model caused limb deficiencies [57] and a TCF1/TCF4 double knockout murine model led to hindgut defects and a gastrointestinal tract transformation [97].

Much of the molecular mechanism regulating IL-6 at the transcriptional level is based on studies in immune cells. Those studies indicate that C/EBP and NF-κB positively regulate IL-6 [34, 36, 86, 98, 99]. One study in astrocytes demonstrated that IL-6 is positively regulated by C/EBP [36]. We also show C/EBP-δ and NF-kB inhibition disturbed IL-6 expression and simultaneously show that β-catenin positively regulates C/EBP-δ and NF-κB. This finding suggests that β-catenin may be a master regulator of IL-6 expression, whereby in presence of its Wnts transcriptional partners, it inhibits promoter activity of IL-6. But in absence of β-catenin, TCFs/LEF, C/EBP, and NF-κB all act independently to activate IL-6 transcription.

Inflammation is not always a negative response. In fact, inflammation is often needed for repair. However, it is hyperinflammation, uncontrolled inflammation that is detrimental. Our data indicate that β-catenin may be a master regulator of NF-κB and C/EBP mediated inflammatory responses. Both NF-κB and C/EBP can be inflammatory through induction of cytokines, which can be either pro or anti-inflammatory in relation to amount and context. As indicated, IL-6 can be either inflammatory or non-inflammatory depending on its signaling pathway and whether it binds to cell associated cognate receptor or soluble receptor. Therefore, we propose that β-catenin is a mean by which to override NF-κB and C/EBP mediated “over activation” and when β-catenin is diminished, there is an unchecked response to NF-κB and C/EBP activity in glial cells that can lead to hyperinflammation.

β-catenin and TCF/LEF may inform novel therapeutic strategies to regulate IL-6 levels in the CNS. Tocilizumab, a humanized anti-IL-6 receptor antibody is now approved for the treatment of rheumatoid arthritis and juvenile idiopathic arthritis [100], but it may not exert an effect in reducing or ameliorating high IL-6 in the CNS in context of neurodegenerative diseases due to poor penetration of antibodies into the CNS. IL-6, however, is not always proinflammatory [20, 45]. It can also exert anti-inflammatory responses through trans-signaling [20, 45]. Therefore, in conditions where IL-6 levels may be needed to be increased within the CNS, to promote repair and survival, inducing TCFs/LEF can be a pathway by which this can occur. In contrast, in conditions where elevated IL-6 is well documented to be associated with neurodegenerative diseases, inhibiting IL-6 may be an approach to reduce and/or ameliorate IL-6 in the CNS. Here, induction of β-catenin may be such an approach. While there are several small molecules that can modulate β-catenin signaling, (https://www.selleckchem.com/Wnt.html), β-catenin signaling is highly context dependent and should be specifically targeted to cell in question. Advances in cell-specific gene therapy may be an approach by which IL-6 expression in the CNS is regulated through β-catenin/TCFs/LEF manipulation to ensure that there are no off-target effects. Continuing to reveal molecular mechanism(s) by which cytokines in the CNS are regulated is critical to reveal their unique regulation in the CNS that may be different than in other organ systems to inform brain-specific targeted strategy to control cytokine-mediated pro- and anti-inflammatory responses in the CNS.

## Conclusions

In summary, we demonstrate a novel and discordant canonical Wnt/β-catenin signaling regulation of IL-6 expression in human astrocytes. Specifically, β-catenin inhibits IL-6 at the transcription level, whereas TCF/LEF partner with other transcription factors to induce IL-6 transcription. β-catenin also regulates NF/κB and c/EBP expression, potentially functioning as a signal that can overcome excessive induction of IL-6 in the brain.

## Supplementary information

**Additional file 1: Supplementary figure 1**. NHAs were evaluated for endogenous expression of IL-6 and GAPDH mRNA by real-time PCR after 48h culture. **Supplementary figure 2.** TCFs/LEF positively regulate IL-6 expression in astrocytes. (a) Endogenous mRNA expression of TCF/LEF family of transcription factors (TFs) in NHAs measured by real-time PCR after 48h culture. **(b)** cDNAs from (Fig. 2b) were used to analyze the TCFs/LEF effects on each other using real-time PCR (*n*=3, one-way ANOVA, with post hoc Dunnett’s test) **Supplementary figure 3.** Characterization of IL-6 promoter reporter plasmid. (a) Astrocytes (U138s) were transfected with p501 (vector control) or IL-6 promoter reporter plasmid and at 48h luciferase assay was performed. (b) U138s transfected with IL-6 promoter plasmid were either unstimulated or stimulated with IL-1β and at 48h luciferase reporter assay was performed. (c) NHAs were transfected with β-catenin or scrm siRNA and at 24h, the cells were transfected with the validated IL-6 reporter plasmid. At 48h post plasmid transfection, luciferase reporter assay was performed. * indicates p≤0.05 in comparison to respective control (n=≥3, one sample T-Test) **Supplementary figure 4.** Endogenous expression of β-catenin, ATF2, Smads (SMAD1,2,3,4,5,6,7,8/9A-B) and Zeb1 mRNAs in NHAs measured by real-time PCR after 48h culture. **Supplementary figure 5.** Endogenous expression of C/EBP and NF-κB family members in NHAs measured by real-time PCR after 48h culture.

## Data Availability

All data generated or analysed during this study are included in this published article and its supplementary information files. The original data supporting these findings are available at any time upon request to the corresponding author.

## Abbreviations

NHA: Normal human astrocytes
IL-6: Interleukin 6
NF-κB: nuclear factor kappa-light-chain-enhancer of activated B cells
C/EBP: CCAAT-enhancer-binding protein
TCFs/LEF: T cell factor/lymphoid enhancing factor
CNS: Central nervous system
AD: Alzheimer’s disease
MS: Multiple Sclerosis
PD: Parkinson’s disease
HD: Huntington’s disease
HAND: HIV-associated neurocognitive disorder
BBB: Blood brain barrier
SASP: Senescence associated secretory phenotype
BIO: 6-bromoindirubin-3′-oxime
TF: Transcription factor
KD: Knockdown
ATF2: Activating transcription factor 2
SMDAD: *Caenorhabditis elegans* SMA (“small” worm phenotype) and Drosophila MAD (“Mothers Against Decapentaplegic”) family of genes
ZEB1: zinc finger E-box binding homeobox 1
PAI: Plasminogen activation inhibitor
EAAT2: Excitatory amino acid transporter 2
GS: Glutamine synthetase

## References

[1] Bauer J, Strauss S, Schreiter-Gasser U, Ganter U, Schlegel P, Witt I, Yolk B, Berger M (1991). Interleukin-6 and α-2-macroglobulin indicate an acute-phase state in Alzheimer's disease cortices. FEBS Lett.

[2] Strauss S, Bauer J, Ganter U, Jonas U, Berger M, Volk B (1992). Detection of interleukin-6 and alpha 2-macroglobulin immunoreactivity in cortex and hippocampus of Alzheimer's disease patients. Lab Invest.

[3] Wood JA, Wood PL, Ryan R, Graff-Radford NR, Pilapil C, Robitaille Y, Quirion R (1993). Cytokine indices in Alzheimer's temporal cortex: no changes in mature IL-1β or IL-1RA but increases in the associated acute phase proteins IL-6, α2-macroglobulin and C-reactive protein. Brain Res.

[4] Hull M, Strauss S, Berger M, Volk B, Bauer J (1996). The participation of interleukin-6, a stress-inducible cytokine, in the pathogenesis of Alzheimer's disease. Behav Brain Res.

[5] Krei K, Fredrikson S, Fontana A, Link H (1991). Interleukin-6 is elevated in plasma in multiple sclerosis. J Neuroimmunol.

[6] Malmeström C, Andersson B, Haghighi S, Lycke J (2006). IL-6 and CCL2 levels in CSF are associated with the clinical course of MS: implications for their possible immunopathogenic roles. J Neuroimmunol.

[7] Mogi M, Harada M, Kondo T, Riederer P, Inagaki H, Minami M, Nagatsu T (1994). Interleukin-1β, interleukin-6, epidermal growth factor and transforming growth factor-α are elevated in the brain from parkinsonian patients. Neurosci Lett.

[8] Nagatsu T, Sawada M: Biochemistry of postmortem brains in Parkinson’s disease: historical overview and future prospects. In Neuropsychiatric Disorders An Integrative Approach. Springer; 2007: 113–120. 10.1007/978-3-211-73574-9_14 17982884

[9] Björkqvist M, Wild EJ, Thiele J, Silvestroni A, Andre R, Lahiri N, Raibon E, Lee RV, Benn CL, Soulet D (2008). A novel pathogenic pathway of immune activation detectable before clinical onset in Huntington's disease. J Exp Med.

[10] Bensadoun JC, De Almeida LP, Dréano M, Aebischer P, Déglon N (2001). Neuroprotective effect of interleukin-6 and IL6/IL6R chimera in the quinolinic acid rat model of Huntington's syndrome. Eur J Neurosci.

[11] Spadaro F, Cecchetti S, Fantuzzi L (2017). Macrophages and phospholipases at the intersection between inflammation and the pathogenesis of HIV-1 infection. Int J Mol Sci.

[12] Clifford DB, Ances BM (2013). HIV-associated neurocognitive disorder. Lancet Infect Dis.

[13] Vartak-Sharma N, Ghorpade A (2012). Astrocyte elevated gene-1 regulates astrocyte responses to neural injury: implications for reactive astrogliosis and neurodegeneration. J Neuroinflammation.

[14] Dowlati Y, Herrmann N, Swardfager W, Liu H, Sham L, Reim EK, Lanctôt KL (2010). A meta-analysis of cytokines in major depression. Biol Psychiatry.

[15] Maes M, Scharpé S, Meltzer HY, Bosmans E, Suy E, Calabrese J, Cosyns P (1993). Relationships between interleukin-6 activity, acute phase proteins, and function of the hypothalamic-pituitary-adrenal axis in severe depression. Psychiatry Res.

[16] Dunn AJ, Swiergiel AH, de Beaurepaire R (2005). Cytokines as mediators of depression: what can we learn from animal studies?. Neurosci Biobehav Rev.

[17] Potvin S, Stip E, Sepehry AA, Gendron A, Bah R, Kouassi E (2008). Inflammatory cytokine alterations in schizophrenia: a systematic quantitative review. Biol Psychiatry.

[18] Patterson PH (2009). Immune involvement in schizophrenia and autism: etiology, pathology and animal models. Behav Brain Res.

[19] Sasayama D, Hattori K, Wakabayashi C, Teraishi T, Hori H, Ota M, Yoshida S, Arima K, Higuchi T, Amano N (2013). Increased cerebrospinal fluid interleukin-6 levels in patients with schizophrenia and those with major depressive disorder. J Psychiatr Res.

[20] Erta M, Quintana A, Hidalgo J (2012). Interleukin-6, a major cytokine in the central nervous system. Int J Biol Sci.

[21] Gruol DL (2015). IL-6 regulation of synaptic function in the CNS. Neuropharmacology.

[22] Oh J, McCloskey MA, Blong CC, Bendickson L, Nilsen-Hamilton M, Sakaguchi DS (2010). Astrocyte-derived interleukin-6 promotes specific neuronal differentiation of neural progenitor cells from adult hippocampus. J Neurosci Res.

[23] Ramesh G, MacLean AG, Philipp MT. Cytokines and chemokines at the crossroads of neuroinflammation, neurodegeneration, and neuropathic pain. Mediat Inflamm. 2013;2013. 10.1155/2013/480739 PMC3753746 23997430

[24] Chakrabarty P, Jansen-West K, Beccard A, Ceballos-Diaz C, Levites Y, Verbeeck C, Zubair AC, Dickson D, Golde TE, Das P (2010). Massive gliosis induced by interleukin-6 suppresses Aβ deposition in vivo: evidence against inflammation as a driving force for amyloid deposition. FASEB J.

[25] Campbell IL, Abraham CR, Masliah E, Kemper P, Inglis JD, Oldstone M, Mucke L (1993). Neurologic disease induced in transgenic mice by cerebral overexpression of interleukin 6. Proc Natl Acad Sci.

[26] Chiang C-S, Stalder A, Samimi A, Campbell IL (1994). Reactive gliosis as a consequence of interleukin-6 expression in the brain: studies in transgenic mice. Dev Neurosci.

[27] Fattori E, Lazzaro D, Musiani P, Modesti A, Alonzi T, Ciliberto G (1995). IL-6 expression in neurons of transgenic mice causes reactive astrocytosis and increase in ramified microglial cells but no neuronal damage. Eur J Neurosci.

[28] Giralt M, Penkowa M, Hernández JN, Molinero A, Carrasco J, Lago N, Camats J, Il C, Hidalgo J (2002). Metallothionein-1+ 2 deficiency increases brain pathology in transgenic mice with astrocyte-targeted expression of interleukin 6. Neurobiol Dis.

[29] Klein MA, Möller JC, Jones LL, Bluethmann H, Kreutzberg GW, Raivich G (1997). Impaired neuroglial activation in interleukin-6 deficient mice. Glia.

[30] Penkowa M, Moos T, Carrasco J, Hadberg H, Molinero A, Bluethmann H, Hidalgo J (1999). Strongly compromised inflammatory response to brain injury in interleukin-6-deficient mice. Glia.

[31] Sugiura S, Lahav R, Han J, Kou SY, Banner LR, De Pablo F, Patterson PH (2000). Leukaemia inhibitory factor is required for normal inflammatory responses to injury in the peripheral and central nervous systems in vivo and is chemotactic for macrophages in vitro. Eur J Neurosci.

[32] Galiano M, Liu ZQ, Kalla R, Bohatschek M, Koppius A, Gschwendtner A, Xu S, Werner A, Kloss CU, Jones LL (2001). Interleukin-6 (IL6) and cellular response to facial nerve injury: effects on lymphocyte recruitment, early microglial activation and axonal outgrowth in IL6-deficient mice. Eur J Neurosci.

[33] Cardenas H, Bolin LM (2003). Compromised reactive microgliosis in MPTP-lesioned IL-6 KO mice. Brain Res.

[34] Ruocco MR, Chen X, Ambrosino C, Dragonetti E, Liu W, Mallardo M, De Falco G, Palmieri C, Franzoso G, Quinto I (1996). Regulation of HIV-1 long terminal repeats by interaction of C/EBP (NF-IL6) and NF-κB/Rel transcription factors. J Biol Chem.

[35] Matsusaka T, Fujikawa K, Nishio Y, Mukaida N, Matsushima K, Kishimoto T, Akira S (1993). Transcription factors NF-IL6 and NF-kappa B synergistically activate transcription of the inflammatory cytokines, interleukin 6 and interleukin 8. Proc Natl Acad Sci.

[36] Poli V (1998). The role of C/EBP isoforms in the control of inflammatory and native immunity functions. J Biol Chem.

[37] Roux P, Alfieri C, Hrimech M, Cohen EA, Tanner JE (2000). Activation of transcription factors NF-kappaB and NF-IL-6 by human immunodeficiency virus type 1 protein R (Vpr) induces interleukin-8 expression. J Virol.

[38] Jirillo E, Covelli V, Brandonisio O, Munno I, De Simone C, Mastroianni CM, Antonaci S, Riccio P (1991). HIV-infection and in vivo lipopolysaccharide-induced release of cytokines. An amplified mechanism of damage to the host. Acta Neurol (Napoli).

[39] Carroll-Anzinger D, Kumar A, Adarichev V, Kashanchi F, Al-Harthi L (2007). Human immunodeficiency virus-restricted replication in astrocytes and the ability of gamma interferon to modulate this restriction are regulated by a downstream effector of the Wnt signaling pathway. J Virol.

[40] Richards MH, Narasipura SD, Kim S, Seaton MS, Lutgen V, Al-Harthi L (2015). Dynamic interaction between astrocytes and infiltrating PBMCs in context of neuroAIDS. Glia.

[41] Yu C, Narasipura SD, Richards MH, Hu XT, Yamamoto B, Al-Harthi L (2017). HIV and drug abuse mediate astrocyte senescence in a beta-catenin-dependent manner leading to neuronal toxicity. Aging Cell.

[42] Henderson LJ, Narasipura SD, Adarichev V, Kashanchi F, Al-Harthi L (2012). Identification of novel T cell factor 4 (TCF-4) binding sites on the HIV long terminal repeat which associate with TCF-4, beta-catenin, and SMAR1 to repress HIV transcription. J Virol.

[43] Narasipura SD, Henderson LJ, Fu SW, Chen L, Kashanchi F, Al-Harthi L (2012). Role of beta-catenin and TCF/LEF family members in transcriptional activity of HIV in astrocytes. J Virol.

[44] Al-Harthi L (2012). Interplay between Wnt/beta-catenin signaling and HIV: virologic and biologic consequences in the CNS. J NeuroImmune Pharmacol.

[45] Rothaug M, Becker-Pauly C, Rose-John S (2016). The role of interleukin-6 signaling in nervous tissue. *Biochimica et Biophysica Acta (BBA)-Molecular*. Cell Res.

[46] Al-Harthi L (2012). Wnt/beta-catenin and its diverse physiological cell signaling pathways in neurodegenerative and neuropsychiatric disorders. J NeuroImmune Pharmacol.

[47] Chinta SJ, Lieu CA, DeMaria M, Laberge RM, Campisi J, Andersen JK (2013). Environmental stress, ageing and glial cell senescence: a novel mechanistic link to Parkinson's disease?. J Intern Med.

[48] Chinta SJ, Woods G, Rane A, Demaria M, Campisi J, Andersen JK (2015). Cellular senescence and the aging brain. Exp Gerontol.

[49] Bhat R, Crowe EP, Bitto A, Moh M, Katsetos CD, Garcia FU, Johnson FB, Trojanowski JQ, Sell C, Torres C (2012). Astrocyte senescence as a component of Alzheimer’s disease. PLoS One.

[50] Tan FC, Hutchison ER, Eitan E, Mattson MP (2014). Are there roles for brain cell senescence in aging and neurodegenerative disorders?. Biogerontology.

[51] Salminen A, Ojala J, Kaarniranta K, Haapasalo A, Hiltunen M, Soininen H (2011). Astrocytes in the aging brain express characteristics of senescence-associated secretory phenotype. Eur J Neurosci.

[52] van Genderen C, Okamura RM, Farinas I, Quo RG, Parslow TG, Bruhn L, Grosschedl R (1994). Development of several organs that require inductive epithelial-mesenchymal interactions is impaired in LEF-1-deficient mice. Genes Dev.

[53] Waterman ML, Fischer WH, Jones KA (1991). A thymus-specific member of the HMG protein family regulates the human T cell receptor C alpha enhancer. Genes Dev.

[54] Oosterwegel M, van de Wetering M, Timmerman J, Kruisbeek A, Destree O, Meijlink F, Clevers H (1993). Differential expression of the HMG box factors TCF-1 and LEF-1 during murine embryogenesis. Development.

[55] van de Wetering M, Oosterwegel M, Dooijes D, Clevers H (1991). Identification and cloning of TCF-1, a T lymphocyte-specific transcription factor containing a sequence-specific HMG box. EMBO J.

[56] Travis A, Amsterdam A, Belanger C, Grosschedl R (1991). LEF-1, a gene encoding a lymphoid-specific protein with an HMG domain, regulates T-cell receptor alpha enhancer function [corrected]. Genes Dev.

[57] Galceran J, Farinas I, Depew MJ, Clevers H, Grosschedl R (1999). Wnt3a−/−−like phenotype and limb deficiency in Lef1(−/−)Tcf1(−/−) mice. Genes Dev.

[58] Roose J, Huls G, van Beest M, Moerer P, van der Horn K, Goldschmeding R, Logtenberg T, Clevers H (1999). Synergy between tumor suppressor APC and the beta-catenin-Tcf4 target Tcf1. Science.

[59] Tang W, Dodge M, Gundapaneni D, Michnoff C, Roth M, Lum L (2008). A genome-wide RNAi screen for Wnt/β-catenin pathway components identifies unexpected roles for TCF transcription factors in cancer. Proc Natl Acad Sci.

[60] Liu F, van den Broek O, Destrée O, Hoppler S (2005). Distinct roles for Xenopus Tcf/Lef genes in mediating specific responses to Wnt/β-catenin signalling in mesoderm development. Development.

[61] Kim CH, Oda T, Itoh M, Jiang D, Artinger KB, Chandrasekharappa SC, Driever W, Chitnis AB (2000). Repressor activity of headless/Tcf3 is essential for vertebrate head formation. Nature.

[62] Merrill BJ, Pasolli HA, Polak L, Rendl M, Garcia-Garcia MJ, Anderson KV, Fuchs E (2004). Tcf3: a transcriptional regulator of axis induction in the early embryo. Development.

[63] Cole MF, Johnstone SE, Newman JJ, Kagey MH, Young RA (2008). Tcf3 is an integral component of the core regulatory circuitry of embryonic stem cells. Genes Dev.

[64] Yi F, Pereira L, Hoffman JA, Shy BR, Yuen CM, Liu DR, Merrill BJ (2011). Opposing effects of Tcf3 and Tcf1 control Wnt stimulation of embryonic stem cell self-renewal. Nat Cell Biol.

[65] Hrckulak D, Kolar M, Strnad H, Korinek V. TCF/LEF transcription factors: an update from the internet resources. Cancers (Basel). 2016;8. 10.3390/cancers8070070 PMC4963812 27447672

[66] Korinek V, Barker N, Moerer P, van Donselaar E, Huls G, Peters PJ, Clevers H (1998). Depletion of epithelial stem-cell compartments in the small intestine of mice lacking Tcf-4. Nat Genet.

[67] Nguyen H, Merrill BJ, Polak L, Nikolova M, Rendl M, Shaver TM, Pasolli HA, Fuchs E (2009). Tcf3 and Tcf4 are essential for long-term homeostasis of skin epithelia. Nat Genet.

[68] Inestrosa NC, Montecinos-Oliva C, Fuenzalida M (2012). Wnt signaling: role in Alzheimer disease and schizophrenia. J NeuroImmune Pharmacol.

[69] Berwick DC, Harvey K. The importance of Wnt signalling for neurodegeneration in Parkinson's disease. Biochem Soc Trans. 2012;40(5):1123–8. 10.1042/BST20120122 22988876

[70] Henderson LJ, Al-Harthi L (2011). Role of beta-catenin/TCF-4 signaling in HIV replication and pathogenesis: insights to informing novel anti-HIV molecular therapeutics. J NeuroImmune Pharmacol.

[71] Park M, Shen K (2012). WNTs in synapse formation and neuronal circuitry. EMBO J.

[72] Lutgen V, Narasipura SD, Sharma A, Min S, Al-Harthi L (2016). beta-catenin signaling positively regulates glutamate uptake and metabolism in astrocytes. J Neuroinflammation.

[73] Fishman D, Faulds G, Jeffery R, Mohamed-Ali V, Yudkin JS, Humphries S, Woo P (1998). The effect of novel polymorphisms in the interleukin-6 (IL-6) gene on IL-6 transcription and plasma IL-6 levels, and an association with systemic-onset juvenile chronic arthritis. J Clin Invest.

[74] Xiao W, Hodge DR, Wang L, Yang X, Zhang X, Farrar WL (2004). NF-kappaB activates IL-6 expression through cooperation with c-Jun and IL6-AP1 site, but is independent of its IL6-NFkappaB regulatory site in autocrine human multiple myeloma cells. Cancer Biol Therapy.

[75] Campisi J (2013). Aging, cellular senescence, and cancer. Annu Rev Physiol.

[76] Kuilman T, Michaloglou C, Vredeveld LC, Douma S, van Doorn R, Desmet CJ, Aarden LA, Mooi WJ, Peeper DS (2008). Oncogene-induced senescence relayed by an interleukin-dependent inflammatory network. Cell.

[77] Sato N, Meijer L, Skaltsounis L, Greengard P, Brivanlou AH (2004). Maintenance of pluripotency in human and mouse embryonic stem cells through activation of Wnt signaling by a pharmacological GSK-3-specific inhibitor. Nat Med.

[78] De Ferrari G, Chacon M, Barria M, Garrido J, Godoy J, Olivares G, Reyes A, Alvarez A, Bronfman M, Inestrosa N (2003). Activation of Wnt signaling rescues neurodegeneration and behavioral impairments induced by β-amyloid fibrils. Mol Psychiatry.

[79] Sprowl S, Waterman ML (2013). Past visits present: TCF/LEFs partner with ATFs for β-catenin–independent activity. PLoS Genet.

[80] Reimold AM, Kim J, Finberg R, Glimcher LH (2001). Decreased immediate inflammatory gene induction in activating transcription factor-2 mutant mice. Int Immunol.

[81] Grumolato L, Liu G, Haremaki T, Mungamuri SK, Mong P, Akiri G, Lopez-Bergami P, Arita A, Anouar Y (2013). Mlodzik M: **β-catenin-independent activation of TCF1/LEF1 in human hematopoietic tumor cells through interaction with ATF2 transcription factors**. PLoS Genet.

[82] Labbé E, Letamendia A, Attisano L (2000). Association of Smads with lymphoid enhancer binding factor 1/T cell-specific factor mediates cooperative signaling by the transforming growth factor-β and Wnt pathways. Proc Natl Acad Sci.

[83] Li Z, Xu Z, Duan C, Liu W, Sun J, Han B (2018). Role of TCF/LEF transcription factors in bone development and osteogenesis. Int J Med Sci.

[84] Yanagisawa M, Nakashima K, Takizawa T, Ochiai W, Arakawa H, Taga T (2001). Signaling crosstalk underlying synergistic induction of astrocyte differentiation by BMPs and IL-6 family of cytokines. FEBS Lett.

[85] Rosmaninho P, Mükusch S, Piscopo V, Teixeira V, Raposo AA, Warta R, Bennewitz R, Tang Y, Herold-Mende C, Stifani S. Zeb1 potentiates genome-wide gene transcription with Lef1 to promote glioblastoma cell invasion. EMBO J. 2018;37. 10.15252/embj.201797115 PMC6068449 29903919

[86] Lawrence T, et al. Cold Spring Harb Perspect Biol. 2009:a001651. 10.1101/cshperspect.a001651 PMC2882124 20457564

[87] Strohmeyer R, Shelton J, Lougheed C, Breitkopf T (2014). CCAAT-enhancer binding protein-β expression and elevation in Alzheimer’s disease and microglial cell cultures. PLoS One.

[88] Li R, Strohmeyer R, Liang Z, Lue LF, Rogers J (2004). CCAAT/enhancer binding protein delta (C/EBPdelta) expression and elevation in Alzheimer's disease. Neurobiol Aging.

[89] Libermann TA, Baltimore D (1990). Activation of interleukin-6 gene expression through the NF-kappa B transcription factor. Mol Cell Biol.

[90] Akira S, Kishimoto T (1992). IL-6 and NF-IL6 in acute-phase response and viral infection. Immunol Rev.

[91] Li W, Henderson LJ, Major EO, Al-Harthi L (2011). IFN-gamma mediates enhancement of HIV replication in astrocytes by inducing an antagonist of the beta-catenin pathway (DKK1) in a STAT 3-dependent manner. J Immunol.

[92] Ma B, Hottiger MO (2016). Crosstalk between Wnt/β-catenin and NF-κB signaling pathway during inflammation. Front Immunol.

[93] Ma B, Fey M, Hottiger MO (2015). WNT/β-catenin signaling inhibits CBP-mediated RelA acetylation and expression of proinflammatory NF-κB target genes. J Cell Sci.

[94] Jang J, Ha J-H, Chung S-I, Yoon Y (2014). β-catenin regulates NF-κB activity and inflammatory cytokine expression in bronchial epithelial cells treated with lipopolysaccharide. Int J Mol Med.

[95] Henderson LJ, Sharma A, Monaco MC, Major EO, Al-Harthi L (2012). Human immunodeficiency virus type 1 (HIV-1) transactivator of transcription through its intact core and cysteine-rich domains inhibits Wnt/beta-catenin signaling in astrocytes: relevance to HIV neuropathogenesis. J Neurosci.

[96] Jia Y, Nie F, Du A, Chen Z, Qin Y, Huang T, Song X, Li L (2014). Thymine DNA glycosylase promotes transactivation of β-catenin/TCFs by cooperating with CBP. J Mol Cell Biol.

[97] Gregorieff A, Grosschedl R, Clevers H (2004). Hindgut defects and transformation of the gastro-intestinal tract in Tcf4(−/−)/Tcf1(−/−) embryos. EMBO J.

[98] Xia C, Cheshire JK, Patel H, Woo P (1997). Cross-talk between transcription factors NF-κB and C/EBP in the transcriptional regulation of genes. Int J Biochem Cell Biol.

[99] Liu T, Zhang L, Joo D, Sun S-C (2017). NF-κB signaling in inflammation. Signal Transduct Target Ther.

[100] Tanaka T, Narazaki M, Kishimoto T (2014). IL-6 in inflammation, immunity, and disease. Cold Spring Harb Perspect Biol.

