# Supplementary material for: β-Catenin and TCFs/LEF signaling discordantly regulate IL-6 expression in astrocytes
Source: Cell Commun Signal. 2020 Jun 16;18:93. doi: 10.1186/s12964-020-00565-2 (PMC7296971; doi:10.1186/s12964-020-00565-2)
Supplement: Supplementary file 2 — Additional file 1: Supplementary figure 1. NHAs were evaluated for endogenous expression of IL-6 and GAPDH mRNA by real-time PCR after 48h culture. Supplementary figure 2. TCFs/LEF positively regulate IL-6 expression in astrocytes. (a) Endogenous mRNA expression of TCF/LEF family of transcription factors (TFs) in NHAs measured by real-time PCR after 48h culture. (b) cDNAs from (Fig. 2b) were used to analyze the TCFs/LEF effects on each other using real-time PCR (n=3, one-way ANOVA, with post hoc Dunnett’s test) Supplementary figure 3. Characterization of IL-6 promoter reporter plasmid. (a) Astrocytes (U138s) were transfected with p501 (vector control) or IL-6 promoter reporter plasmid and at 48h luciferase assay was performed. (b) U138s transfected with IL-6 promoter plasmid were either unstimulated or stimulated with IL-1β and at 48h luciferase reporter assay was performed. (c) NHAs were transfected with β-catenin or scrm siRNA and at 24h, the cells were transfected with the validated IL-6 reporter plasmid. At 48h post plasmid transfection, luciferase reporter assay was performed. * indicates p≤0.05 in comparison to respective control (n=≥3, one sample T-Test) Supplementary figure 4. Endogenous expression of β-catenin, ATF2, Smads (SMAD1,2,3,4,5,6,7,8/9A-B) and Zeb1 mRNAs in NHAs measured by real-time PCR after 48h culture. Supplementary figure 5. Endogenous expression of C/EBP and NF-κB family members in NHAs measured by real-time PCR after 48h culture. [file 12964_2020_565_MOESM2_ESM.pptx]

## Slide 1
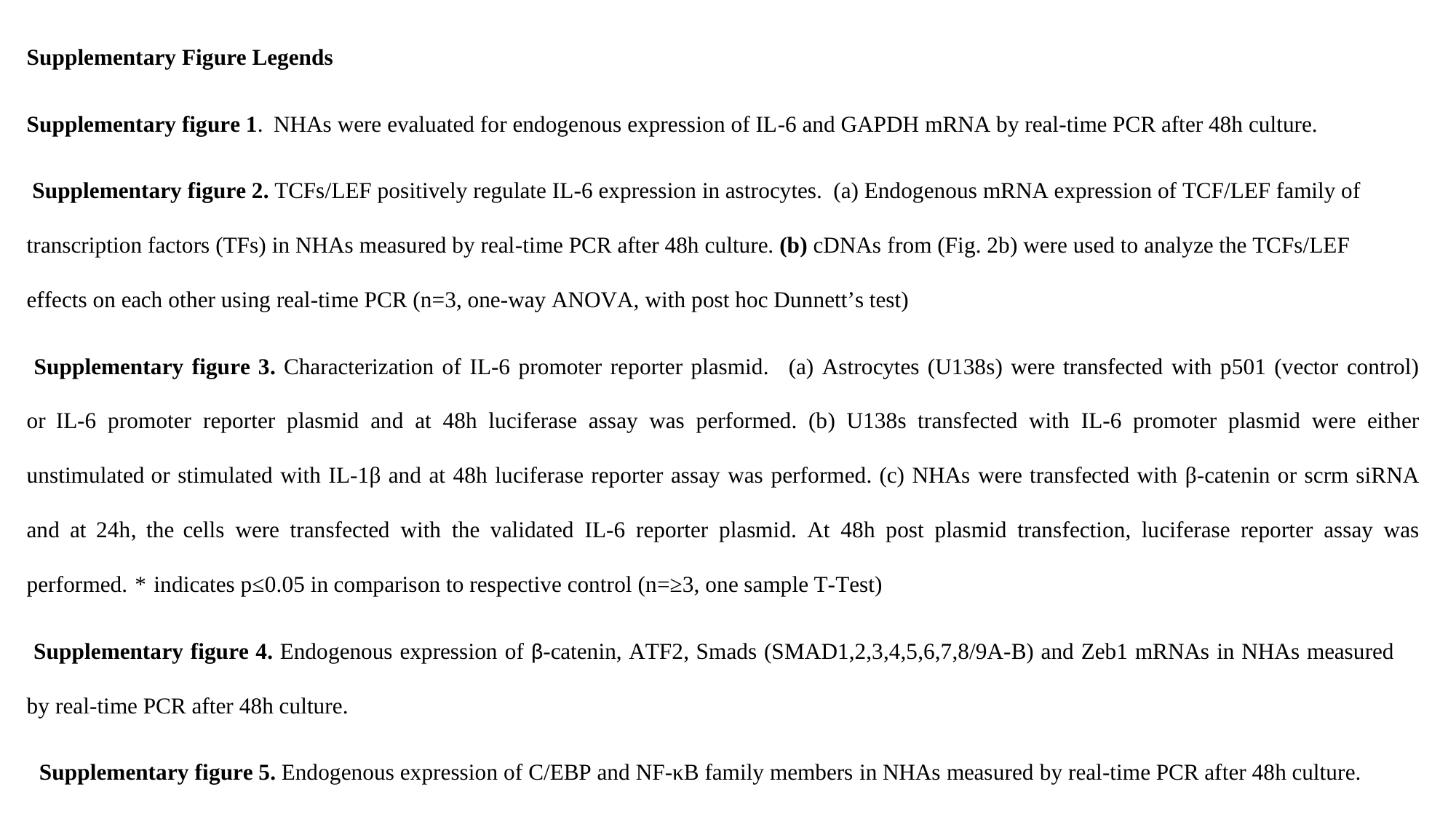

Supplementary Figure Legends
Supplementary figure 1. NHAs were evaluated for endogenous expression of IL-6 and GAPDH mRNA by real-time PCR after 48h culture.
Supplementary figure 2. TCFs/LEF positively regulate IL-6 expression in astrocytes. (a) Endogenous mRNA expression of TCF/LEF family of transcription factors (TFs) in NHAs measured by real-time PCR after 48h culture. (b) cDNAs from (Fig. 2b) were used to analyze the TCFs/LEF effects on each other using real-time PCR (n=3, one-way ANOVA, with post hoc Dunnett’s test)
Supplementary figure 3. Characterization of IL-6 promoter reporter plasmid. (a) Astrocytes (U138s) were transfected with p501 (vector control) or IL-6 promoter reporter plasmid and at 48h luciferase assay was performed. (b) U138s transfected with IL-6 promoter plasmid were either unstimulated or stimulated with IL-1β and at 48h luciferase reporter assay was performed. (c) NHAs were transfected with β-catenin or scrm siRNA and at 24h, the cells were transfected with the validated IL-6 reporter plasmid. At 48h post plasmid transfection, luciferase reporter assay was performed. * indicates p≤0.05 in comparison to respective control (n=≥3, one sample T-Test)
Supplementary figure 4. Endogenous expression of β-catenin, ATF2, Smads (SMAD1,2,3,4,5,6,7,8/9A-B) and Zeb1 mRNAs in NHAs measured by real-time PCR after 48h culture.
Supplementary figure 5. Endogenous expression of C/EBP and NF-κB family members in NHAs measured by real-time PCR after 48h culture.

## Slide 2
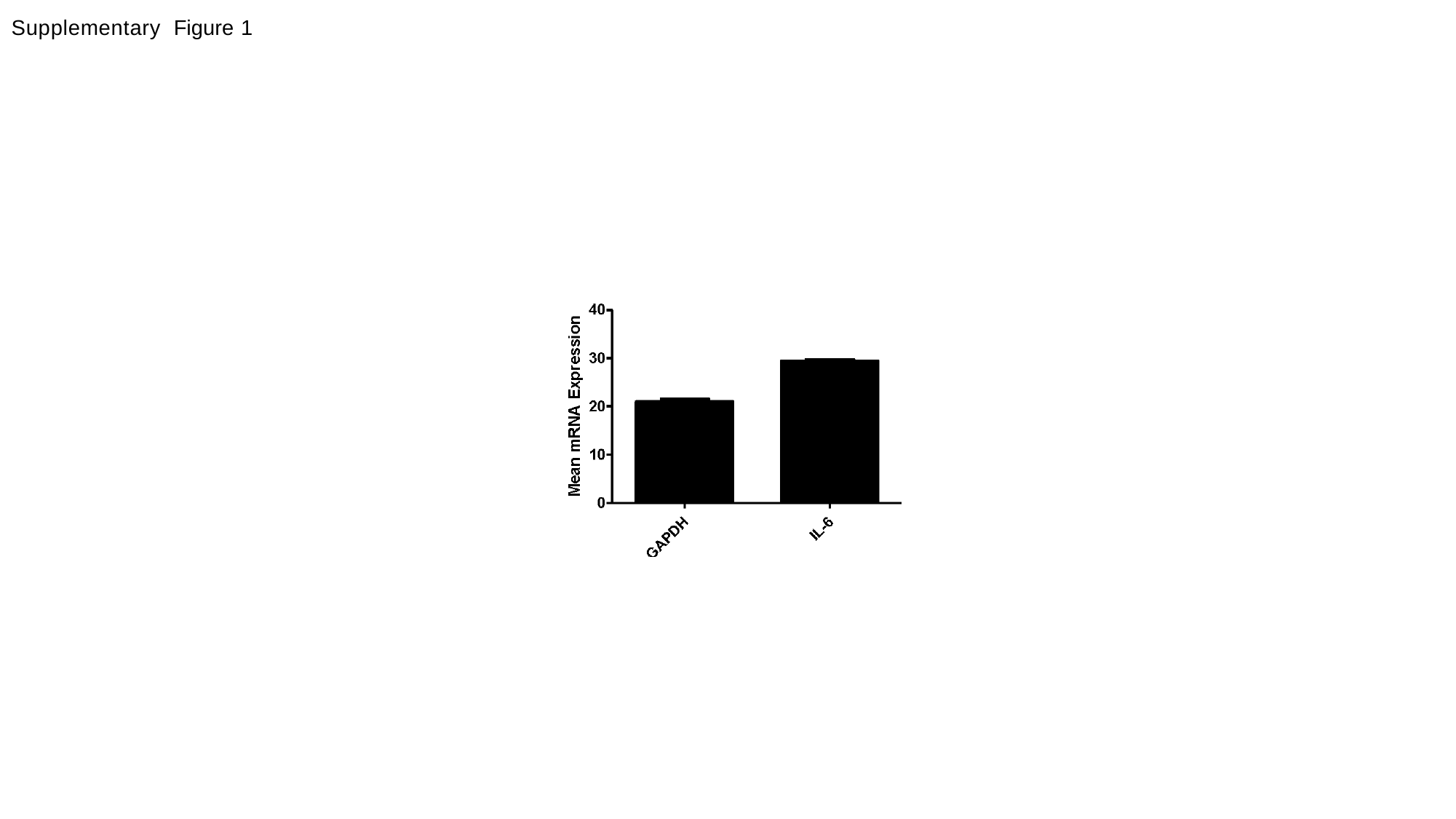

Supplementary Figure 1

## Slide 3
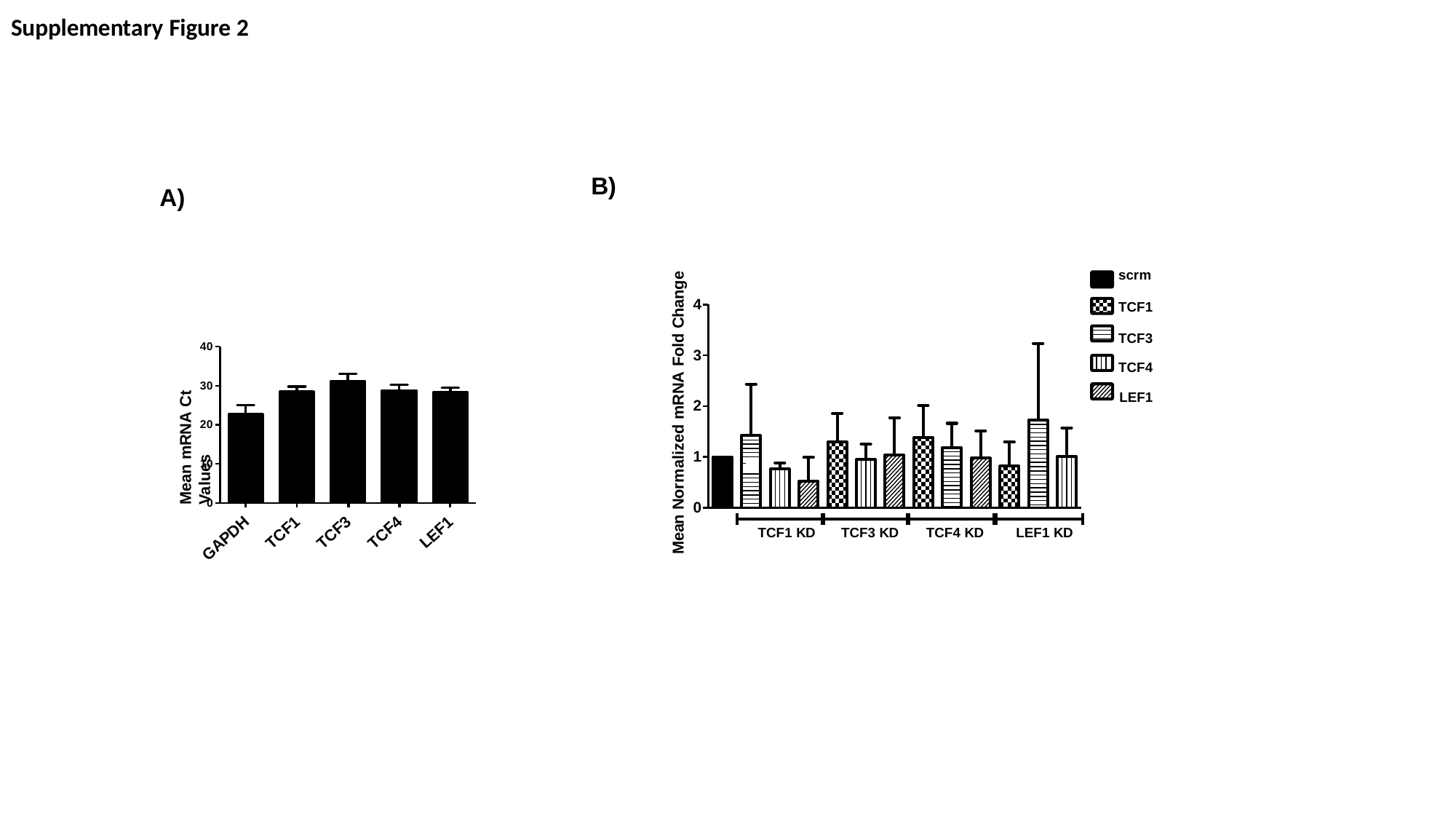

Supplementary Figure 2
B)
A)
Mean Normalized mRNA Fold Change
scrm
4
TCF1
TCF3
40
Mean mRNA Ct Values
3
TCF4
30
LEF1
2
20
1
10
0
0
LEF1
TCF3
TCF1
TCF4
TCF1 KD
TCF3 KD
TCF4 KD
LEF1 KD
GAPDH

## Slide 4
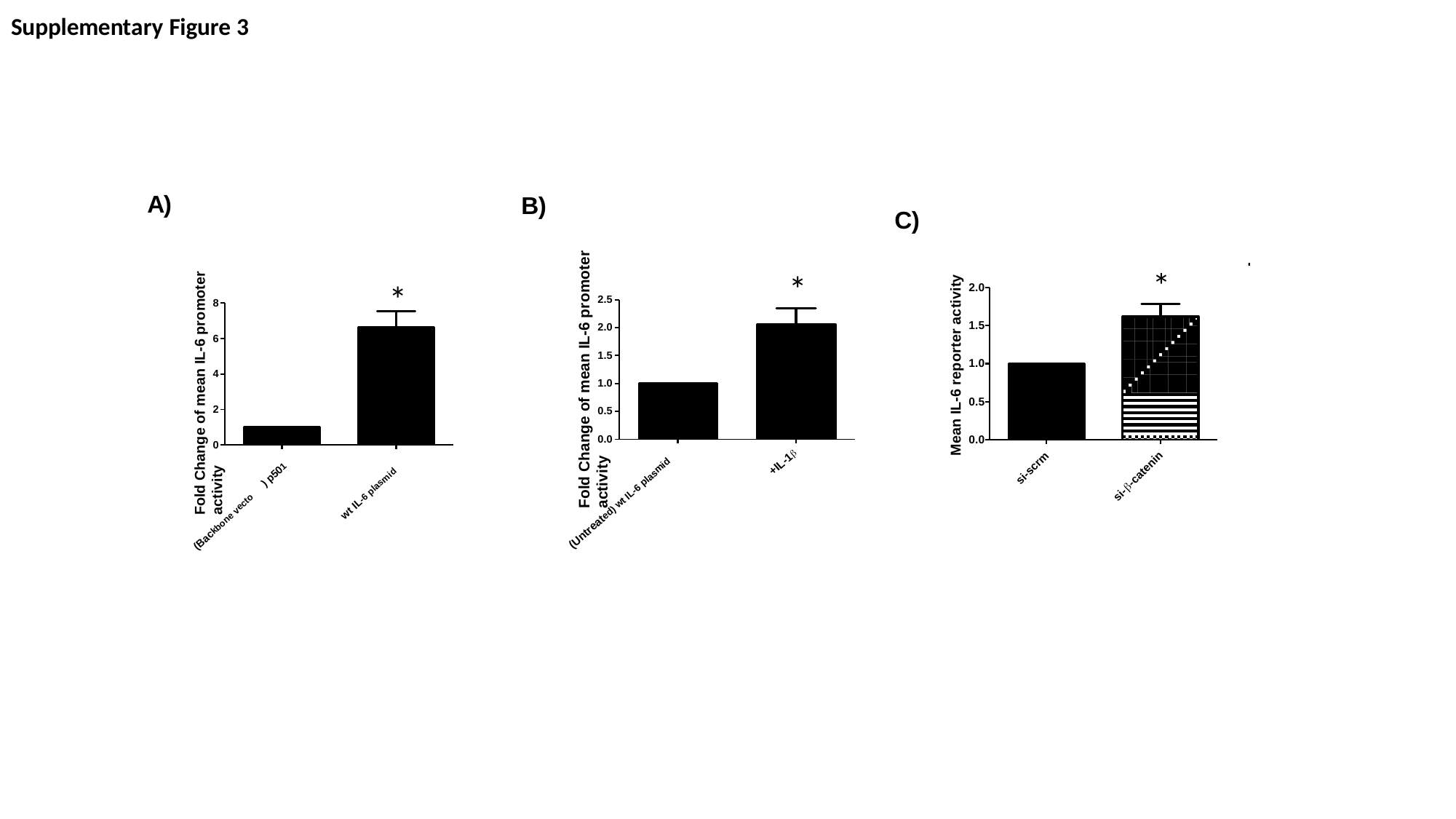

Supplementary Figure 3
A)
B)
C)
Fold Change of mean IL-6 promoter activity
Fold Change of mean IL-6 promoter activity
*
*
Mean IL-6 reporter activity
*
2.0
2.5
8
1.5
2.0
6
1.5
1.0
4
1.0
0.5
2
0.5
0.0
0.0
0
+IL-1β
si-scrm
) p501
si-β-catenin
wt IL-6 plasmid
(Untreated) wt IL-6 plasmid
(Backbone vecto

## Slide 5
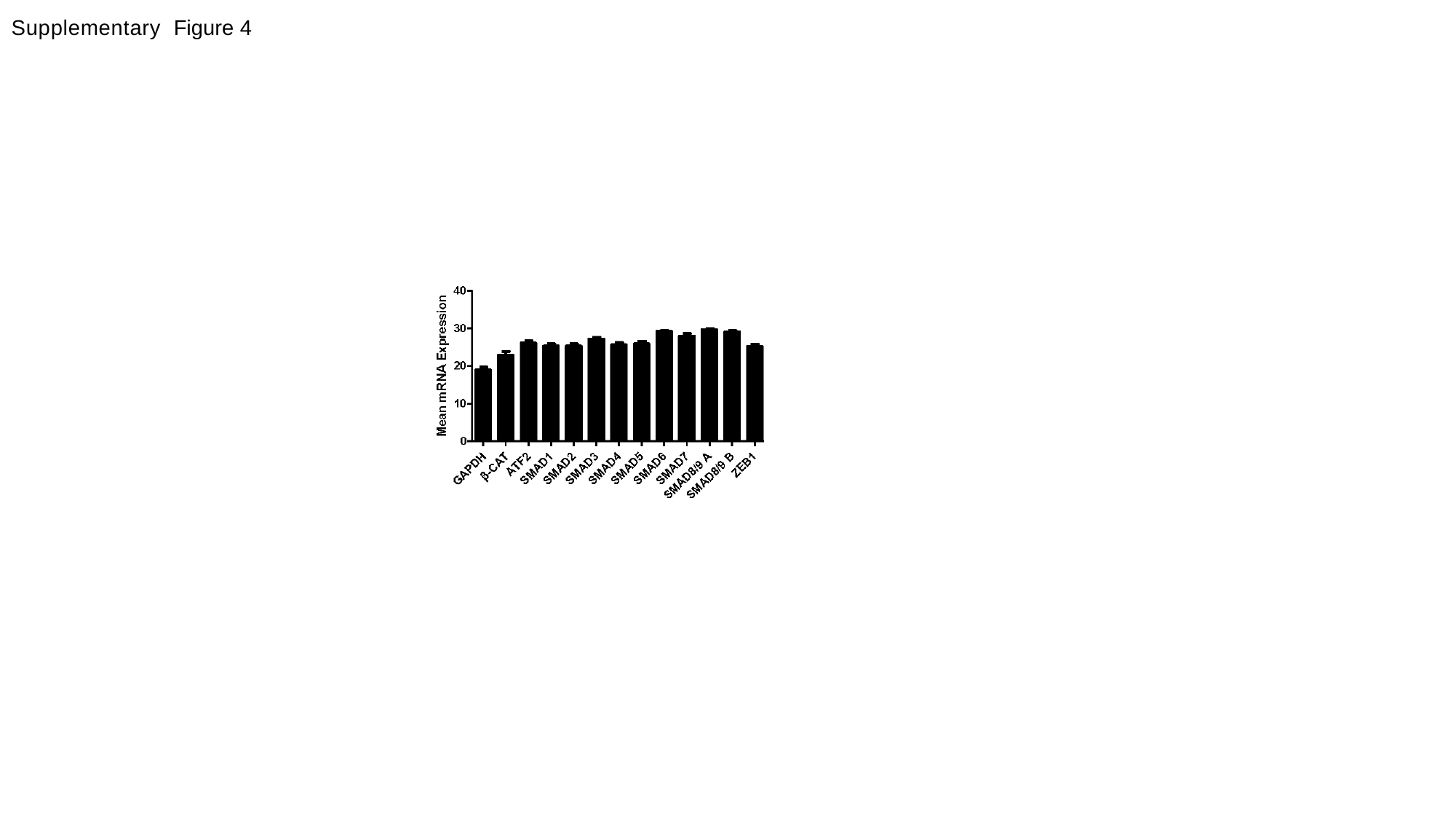

Supplementary Figure 4

## Slide 6
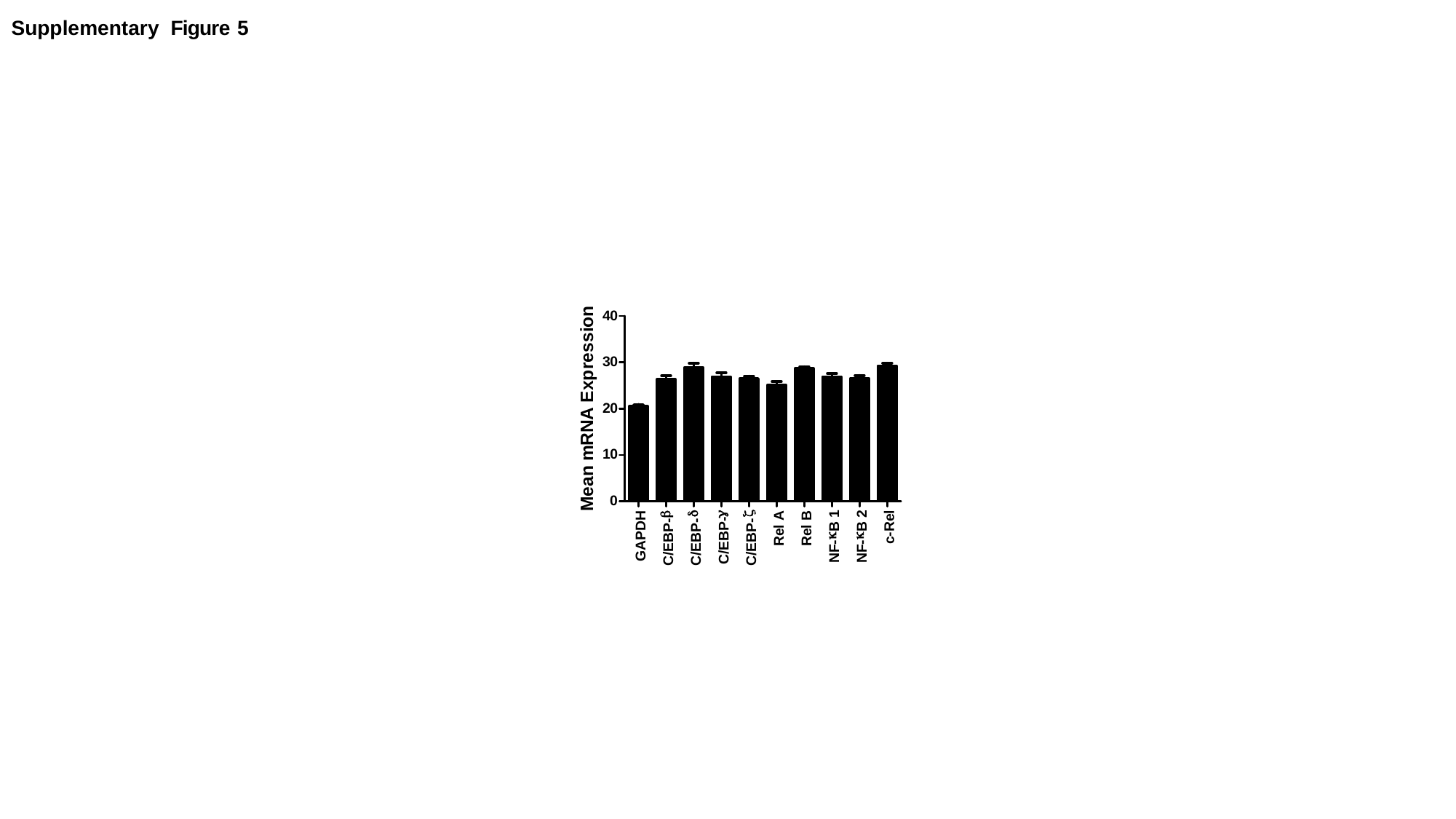

Supplementary Figure 5
